# Predicting gastric cancer response to anti-HER2 therapy or anti-HER2 combined immunotherapy based on multi-modal data

**DOI:** 10.1038/s41392-024-01932-y

**Published:** 2024-08-26

**Authors:** Zifan Chen, Yang Chen, Yu Sun, Lei Tang, Li Zhang, Yajie Hu, Meng He, Zhiwei Li, Siyuan Cheng, Jiajia Yuan, Zhenghang Wang, Yakun Wang, Jie Zhao, Jifang Gong, Liying Zhao, Baoshan Cao, Guoxin Li, Xiaotian Zhang, Bin Dong, Lin Shen

**Affiliations:** 1 Center for Data Science, Peking University, Beijing, China; 2 Department of Gastrointestinal Oncology, Key Laboratory of Carcinogenesis and Translational Research (Ministry of Education), Peking University Cancer Hospital and Institute, Beijing, China; 3 Department of Pathology, Key Laboratory of Carcinogenesis and Translational Research (Ministry of Education), Peking University Cancer Hospital and Institute, Beijing, China; 4 Department of Radiology, Key Laboratory of Carcinogenesis and Translational Research (Ministry of Education), Peking University Cancer Hospital and Institute, Beijing, China; 5 National Biomedical Imaging Center, Peking University, Beijing, China; 6 Department of General Surgery, Nanfang Hospital, Southern Medical University, Guangzhou, China; 7 Guangdong Provincial Key Laboratory of Precision Medicine for Gastrointestinal Tumor, Guangzhou, China; 8 Department of Medical Oncology and Radiation Sickness, Peking University Third Hospital, Beijing, China; 9 National Engineering Laboratory for Big Data Analysis and Applications, Peking University, Beijing, China; 10 Beijing International Center for Mathematical Research (BICMR), Peking University, Beijing, China; 11 Center for Machine Learning Research, Peking University, Beijing, China

**Keywords:** Gastrointestinal cancer, Translational research, Gastrointestinal cancer

## Abstract

The sole use of single modality data often fails to capture the complex heterogeneity among patients, including the variability in resistance to anti-HER2 therapy and outcomes of combined treatment regimens, for the treatment of HER2-positive gastric cancer (GC). This modality deficit has not been fully considered in many studies. Furthermore, the application of artificial intelligence in predicting the treatment response, particularly in complex diseases such as GC, is still in its infancy. Therefore, this study aimed to use a comprehensive analytic approach to accurately predict treatment responses to anti-HER2 therapy or anti-HER2 combined immunotherapy in patients with HER2-positive GC. We collected multi-modal data, comprising radiology, pathology, and clinical information from a cohort of 429 patients: 310 treated with anti-HER2 therapy and 119 treated with a combination of anti-HER2 and anti-PD-1/PD-L1 inhibitors immunotherapy. We introduced a deep learning model, called the Multi-Modal model (MuMo), that integrates these data to make precise treatment response predictions. MuMo achieved an area under the curve score of 0.821 for anti-HER2 therapy and 0.914 for combined immunotherapy. Moreover, patients classified as low-risk by MuMo exhibited significantly prolonged progression-free survival and overall survival (log-rank test, *P* < 0.05). These findings not only highlight the significance of multi-modal data analysis in enhancing treatment evaluation and personalized medicine for HER2-positive gastric cancer, but also the potential and clinical value of our model.

## Introduction

Gastric cancer (GC) is the fifth most prevalent cancer globally, and the second most common cancer in China.^1^ Approximately 15–30% of advanced gastric or gastroesophageal junction adenocarcinomas exhibit amplification or overexpression of the human epidermal growth factor receptor 2 (ERBB2/HER2).^2^ The heterogeneity of this biomarker poses substantial challenges for effective treatment, with responses varying widely among patients. The trastuzumab for GC trial revealed that less than half patients with HER2-positive responded to a combination of trastuzumab and chemotherapy,^3^ indicating significant intra-patient and inter-tumor variability. Further complicating the treatment landscape, the KEYNOTE-811 study’s interim findings^4^ showed that although adding pembrolizumab to standard therapy substantially increases objective response rates as a first-line therapy, this does not equate to a uniform enhancement in overall survival (OS) for all patients. The discrepancies in survival rates underscore the complexity of the disease and suggest that conventional monomodal data may be insufficient for understanding the diverse presentations of HER2-positive GC, necessitating a comprehensive evaluation using multi-modal data. By integrating clinical profiles, radiological imaging, and pathological samples, a more nuanced understanding of tumor behavior is possible, which is imperative for refining treatment decisions. Therefore, an integrated multi-modal approach is essential: to fully characterize the heterogeneity of HER2-positive GC and devise personalized and effective treatment strategies.

The potential of artificial intelligence (AI) as an innovative tool for developing multimodal models is high,^5–9^ and its strength lies in its ability to analyze different data types and integrate them at the feature level.^10–16^ However, the application of AI in predicting treatment response is still in its infancy,^17^ particularly for predicting treatment response in complex diseases such as GC,^18,19^ a task that is far more difficult than diagnosis.^20^ Diagnostic models typically rely on large datasets, including normal and abnormal samples.^20,21^ However, predicting treatment response requires more refined datasets that are specific to a particular disease stage and that reflect the subtle effects of different treatment regimens over time.^22,23^ Additionally, the incompleteness of treatment datasets poses technical challenges in the AI model construction and learning process.^24,25^ In realistic anti-HER2 therapy or anti-HER2 combined immunotherapy scenarios, the patient’s imaging history may only be partially available. This modality deficit has not been fully considered in many studies,^26,27^ while it may seriously affect the learning ability of the model and its utility in clinical decision-making.

To address the challenges, this study aimed to use a comprehensive analytical approach to accurately predict the treatment response in patients with GC receiving anti-HER2 therapy or anti-HER2 combined immunotherapy. We assembled a comprehensive multi-center dataset of 429 patients. This valuable dataset integrates diverse modalities of information from the baseline treatment phase, including radiological computerized tomography (CT) scans, pathological whole-slide images, radiological and pathological reports, and general patient information. Our study focused on two distinct cohorts of patients treated at the Peking Cancer Hospital: the anti-HER2 (those receiving anti-HER2 therapy and chemotherapy) and anti-HER2 combined immunotherapy (those receiving anti-HER2 therapy combined with anti-PD-1/PD-L1 immune checkpoint inhibitors [ICI] and chemotherapy) cohorts. We further supplemented these cohorts with additional patients from external medical facilities. With this rich dataset, we developed a unified, transformer-based deep learning model called the **Mu**lti-**Mo**dal model (MuMo), which effectively incorporates multi-modal inputs to predict the treatment response. MuMo represents a major advancement in leveraging diverse data types, even in cases of missing modalities, to improve prediction accuracy. Experimental results demonstrate MuMo’s ability to extract complementary insights from multi-modal data and provide more accurate treatment response predictions.

## Results

### Multi-modal dataset and cohort characteristics in HER2-positive GC study

**Fig. 1:**
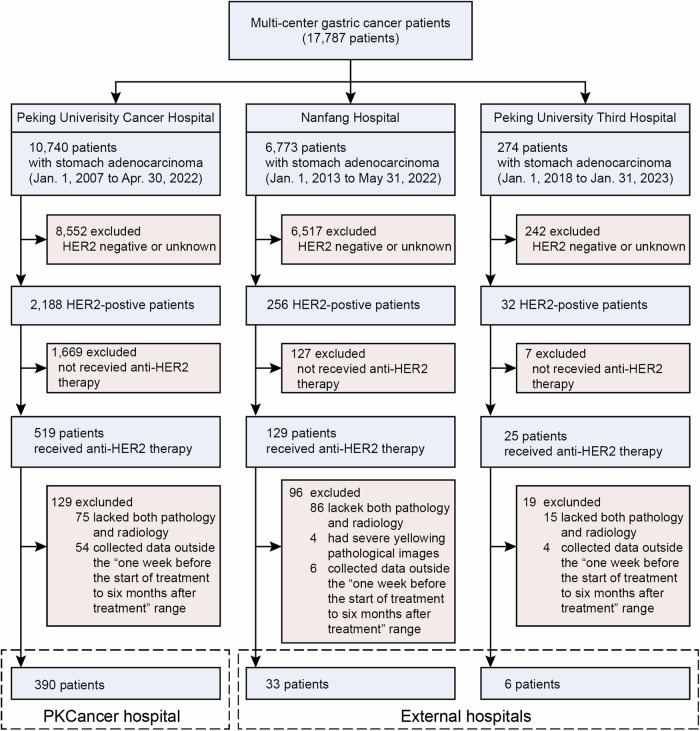
Multi-modal data collection and filtering flowchart for patients with HER2-positive GC from multi-center study. The cohort, comprising 17,787 patients with GC, was derived from a consecutive series of patients diagnosed with stomach adenocarcinoma at Peking University Cancer (PKCancer) Hospital (10,740 patients), Nanfang Hospital (6773 patients), and Peking University Third Hospital (274 patients) between January 2007 and January 2023. The selection process involved exclusions based on HER2 negativity or unknown status, absence of anti-HER2 therapy, and lack of comprehensive multimodal data, including necessary pathological and radiological information collected within a defined period around treatment initiation. The final multi-modal analysis cohort was segmented into 390 patients from the PKCancer Hospital and 39 patients from external hospitals

**Fig. 2:**
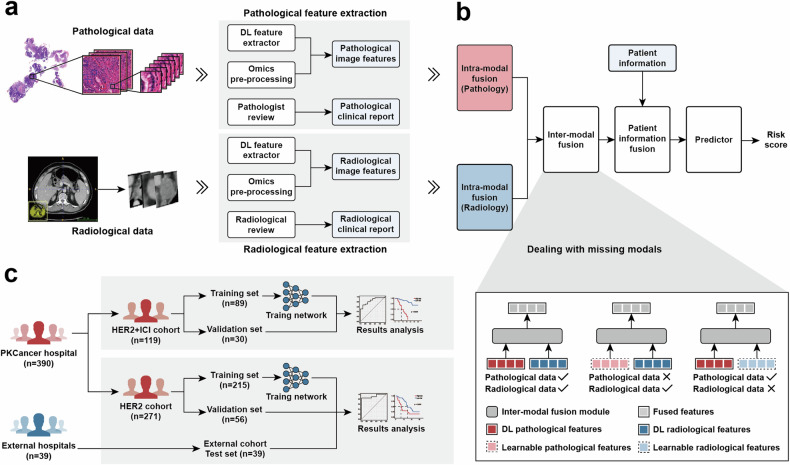
Workflow of the Multi-Modal Model (MuMo) for predicting treatment response to patients with anti-HER2 GC. **a** Feature extraction process: Pathological WSIs and radiological CT scans were processed to extract deep and omics features, which were correlated with clinical reports provided by pathologists or radiologists. **b** Multi-modal information fusion process: MuMo employs intra-modal fusion modules to integrate image features and clinical reports from pathology and radiology to obtain enhanced features. These features were then amalgamated using an inter-modal fusion module, and the patient information was incorporated using a separate patient information fusion module. Subsequently, a predictor was used to predict the risk scores. MuMo can handle missing modalities by employing learnable modality features as placeholders. **c** Overview of experimental pipeline: Data were sourced from Beijing Cancer Hospital (PKCancer) and external hospitals. The patients were divided into anti-HER2 and anti-HER2 combined immunotherapy, as well as external cohorts. The anti-HER2 cohort was randomly divided into a training set to train the model and a validation set to tune its parameters. The final model with frozen parameters was used to analyze the results. Additionally, an external cohort was used as an independent test set to test the robustness of the model. In the anti-HER2 combined immunotherapy cohort, a similar analytical process was employed

Our study commenced with collecting an extensive multi-modal dataset encompassing radiology, pathology, and patient information, from a large group of 17,787 patients with GC during the baseline treatment phase at multiple centers. This dataset included data from Peking University Cancer (PKCancer) Hospital, Nanfang Hospital, and Peking University Third Hospital. Rigorous selection criteria were applied to refine the cohort (Fig. 1). These criteria include excluding patients with negative or unknown HER2 status, those not undergoing anti-HER2 therapy, and those lacking multi-modal data. This process resulted in a cohort of 429 patients with HER2-positive GC, between January 2007 and January 2023 (Table 1) (with additional information in Supplementary Tables S1, S2). As shown in Fig. 2, of the 429 patients, 390 were from Peking University Cancer Hospital. Among them, 271 underwent anti-HER2 therapy, forming an anti-HER2 cohort. The remaining 119 patients, forming an anti-HER2 combined immunotherapy cohort, received a combination of anti-HER2 therapy with either anti-PD-1 inhibitors (85 patients) or anti-PD-L1 inhibitors (34 patients). Additionally, we included an external cohort of 39 patients from Nanfang Hospital and Peking University Third Hospital. Most patients in these cohorts were diagnosed with stage IV GC, with a prevalence of 98.52% in the anti-HER2 cohort, 97.48% in the anti-HER2 combined immunotherapy cohort, and 97.44% in the external cohort. The median age of the patients in the three cohorts was 63 (interquartile range [IQR]: 55–69), 65 (IQR: 58–72), and 60 years (IQR: 55–68), respectively. The percentage of men was 83.03% in the anti-HER2 cohort, 79.83% in the anti-HER2 combined immunotherapy cohort, and 76.92% in the external cohort. Regarding tumor location, the majority were non-gastroesophageal junction (non-GEJ) tumors, accounting for 69.74%, 70.59%, and 79.49% of patients in each cohort, respectively. Additionally, most patients in all cohorts had either moderately differentiated (47.60%, 47.06%, and 35.90%, respectively) or poorly differentiated (48.71%, 52.10%, and 58.97%, respectively) carcinomas.

**Table 1:** Baseline characteristics of anti-HER2, anti-HER2 combined immunotherapy and external hospital cohorts

Characteristic	Anti-HER2 cohort (*n* = 271)	Anti-HER2 combined immunotherapy cohort (*n* = 119)	External cohort (*n* = 39)
Age			
Median, IQR	63, 55–69	65, 58–72	60, 55–68
Sex			
Male	225 (83.03%)	95 (79.83%)	30 (76.92%)
Female	46 (16.97%)	24 (20.17%)	9 (23.08%)
Tumor site			
GEJ	82 (30.26%)	35 (29.41%)	8 (20.51%)
Non-GEJ	189 (69.74%)	84 (70.59%)	31 (79.49%)
Degree of differentiation			
Poorly	132 (48.71%)	62 (52.10%)	23 (58.97%)
Moderately	129 (47.60%)	56 (47.06%)	14 (35.90%)
Well	10 (3.69%)	1 (0.84%)	2 (5.13%)
Lauren type			
Intestinal	17 (63.84%)	82 (68.91%)	6 (15.79%)
Diffused	34 (12.55%)	12 (10.08%)	1 (2.63%)
Mixed	43 (15.87%)	16 (13.45%)	1 (2.63%)
N/A	21 (7.75%)	9 (7.56%)	31 (79.49%)
PD-L1 expression			
Positive	44 (16.24%)	43 (36.13%)	8 (20.51%)
Negative	60 (22.14%)	25 (21.01%)	8 (20.51%)
N/A	167 (61.62%)	51 (42.86%)	23 (58.97%)
MMR status			
pMMR	137 (50.55%)	104 (87.39%)	18 (46.15%)
dMMR	2 (0.74%)	1 (0.84%)	0 (0.00%)
N/A	132 (48.71%)	14 (11.76%)	21 (53.85%)
EBV status			
Positive	3 (1.11%)	1 (0.84%)	0 (0.00%)
Negative	119 (43.91%)	89 (74.79%)	9 (23.08%)
N/A	149 (54.98%)	29 (24.37%)	30 (76.92%)
TNM stages			
III	4 (1.48%)	3 (2.52%)	1 (2.56%)
IV	267 (98.52%)	116 (97.48%)	38 (97.44%)

**Fig. 3:**
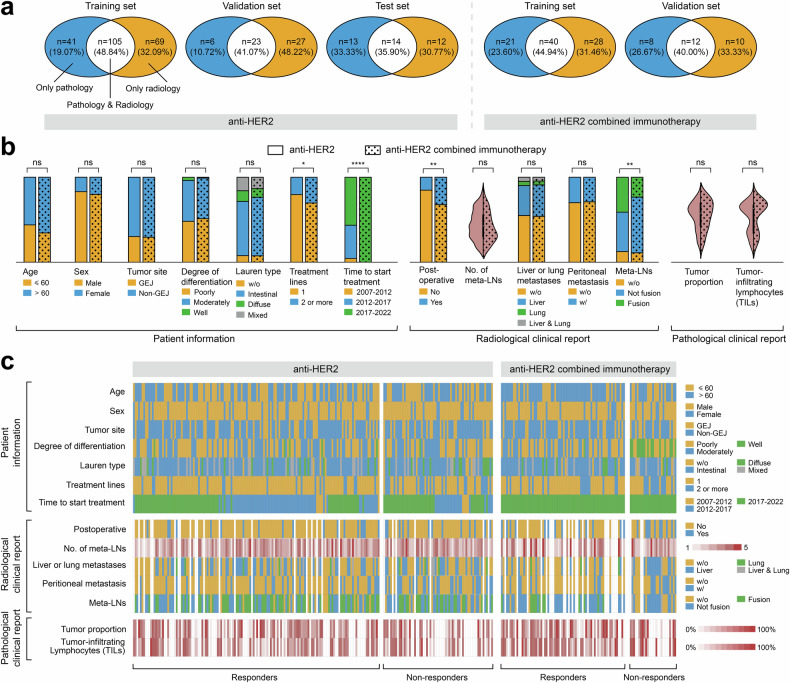
Data characteristics of anti-HER2 and anti-HER2 combined immunotherapy cohorts. **a** Distribution of proportions for pathological and radiological image data across different sets. **b** Distribution of clinical information, encompassing patient information, radiological clinical reports, and pathological clinical reports. Stacked bars and violin plots were used for discrete and continuous data, respectively. **c** Heatmap illustrating the distribution of clinical information at the individual level, with each row representing one piece of clinical information and each column representing one individual. Some clinical information items with multiple labels, such as the location of the metastatic lymph nodes, HER2 expression heterogeneity, and tumor locations, are presented in Supplementary Fig. S1

Our amassed multi-modal dataset offers rich and comprehensive patient data. This included demographic details (that is, age and sex), tumor characteristics (that is, tumor location, degree of differentiation, and Lauren classification), and treatment specifics (that is, lines of treatment received and time elapsed before initiating treatment). These details are visually represented in Fig. 3b, c and Supplementary Fig. S1. Moreover, each patient’s dataset included data from at least one modality of both pathology and radiology. However, complete data from both modalities (radiology and pathology) was available for less than half of the patients (Fig. 3a). To address this variability in data availability, learnable embeddings were introduced as placeholders for missing modalities. This technology helps to infer missing information, enhancing the robustness of our multi-modal model (detailed methodology in the methods section). Furthermore, structured clinical reports from both radiological and pathological assessments provided additional crucial clinical insights, aiding in a more comprehensive understanding of each patient’s condition (Fig. 2b, c and Supplementary Fig. S1). Specifically, radiological reports included detailed information on post-operative status (whether the patient had undergone gastrectomy), the count and locations of metastatic lymph nodes, occurrences of liver or lung metastases, peritoneal metastasis, and diversity in metastatic lymph node types. In contrast, pathological reports encompassed data on the proportion of tumors, tumor-infiltrating lymphocytes (TILs), and variability in HER2 expression within the tumor.

### MuMo’s predictive performance in the anti-HER2 cohort

**Fig. 4:**
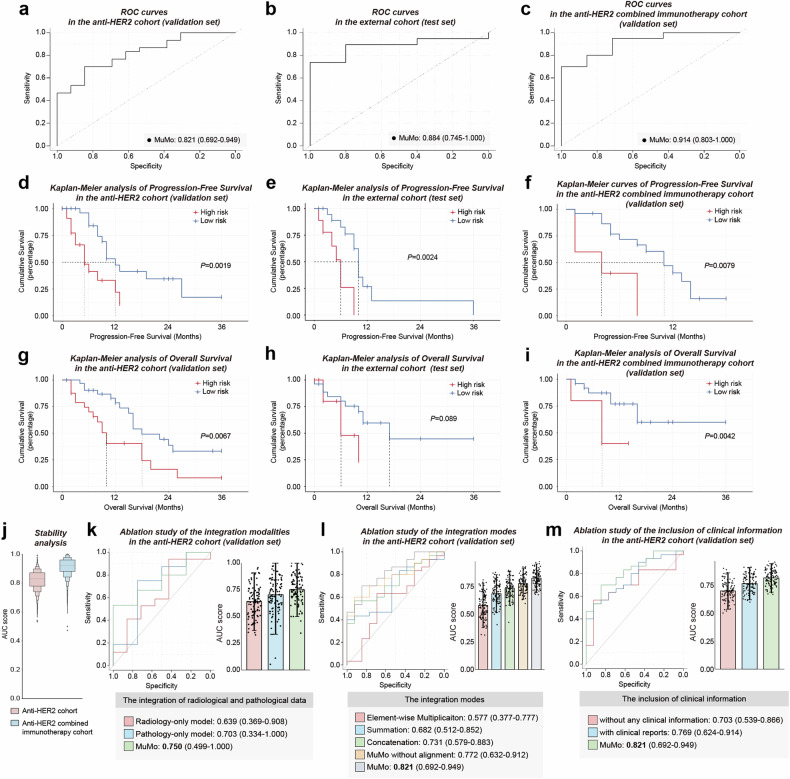
Performance of the Multi-Modal Model (MuMo). **a**–**c** Receiver operating characteristic (ROC) curves display MuMo’s performance in predicting treatment responses, distinguishing between non-responders and responders. These curves pertain to the validation and test sets of the anti-HER2 cohort and the validation set of the anti-HER2 combined immunotherapy cohort. **d**–**f** Kaplan**–**Meier (KM) curves depict Progression-Free Survival (PFS) based on MuMo predictions. These curves were derived from the validation and test sets of the anti-HER2 cohort and the validation set of the anti-HER2 combined immunotherapy cohort. In the Kaplan–Meier analysis, patients were categorized into high-risk (red line) and low-risk (blue line) groups using the Youden index. The log-rank (Mantel-Cox) test was used to determine statistical significance, with a two-sided *P*-value of < 0.05 set as significant. **g**–**i** Kaplan–Meier (KM) curves show Overall Survival (OS) based on MuMo predictions for the validation and test sets of the anti-HER2 cohort and the validation set of the anti-HER2 combined immunotherapy cohort. **j** Stability analysis of MuMo in treatment response prediction. Ablation studies evaluating the integration of radiological and pathological data (**k**), the impact of various integration modes (**l**), and the inclusion of clinical information within MuMo (**m**). Error bars represent the 95% confidence intervals (CI) for the AUC scores

The proposed MuMo demonstrated promising efficacy in predicting treatment responses in the anti-HER2 cohort, achieving an area under the curve (AUC) score of 0.821 (95% Confidence Interval [CI]: 0.692–0.949; Fig. 4a and Supplementary Table S3). Additionally, MuMo exhibited an impressive number needed to treat a value of 1.83 (95% CI: 1.28–4.24; refer to Supplementary Table S4), indicating a high efficiency in predicting treatment response. MuMo’s predictive performance surpassed that of the six individual clinicians in a similar test and even matched the combined score of consultation among these clinicians (Supplementary Figs. S2a, b). To evaluate the generalizability of MuMo, we applied it to an external cohort that functioned as an independent test set. MuMo showed a strong discriminative ability in distinguishing non-responders from responders, as indicated by an AUC score of 0.884 (95% CI: 0.745–1.000; Fig. 4b). This performance highlights the effectiveness of MuMo in multi-center data cases. Additionally, we have showcased the flexible extensibility of the MuMo framework by utilizing two public datasets: TCGA-STAD for gastric adenocarcinoma and TCGA-BRCA for invasive breast carcinoma, detailed in Supplementary Figs. S3, S4 and Supplementary Text S1. This extensibility is further illustrated through its application to potential molecular pathology data, as documented in Supplementary Fig. S5 and Supplementary Text S2. Using the Youden index, a statistical measure derived from the receiver operating characteristic (ROC) curves, we stratified each cohort into high- and low-risk groups based on MuMo’s predictive scores (see Supplementary Text S3 for details). The low-risk group exhibited significantly longer progression-free survival (PFS) (log-rank test, *P* = 0.0019 in the validation set and *P* = 0.0024 in the test set; Fig. 4d, e) and increased OS (log-rank test, *P* = 0.0067 in the validation set; Fig. 4g) than the high-risk group. Notably, in the independent test set, a marked difference in median OS was observed between the two groups (6 months for the high-risk group vs. 17 months for the low-risk group; Fig. 4h).

### MuMo’s adaptability in predicting responses in the anti-HER2 combined immunotherapy cohort

To further evaluate the adaptability of MuMo in diverse treatment cohorts, we analyzed its predictive efficacy in the anti-HER2 combined immunotherapy cohort. In this cohort, MuMo demonstrated remarkable performance with an AUC of 0.914 (95% CI: 0.803–1.000; Fig. 4c, Supplementary Table S3 and Supplementary Fig. S2c), indicating high accuracy in response predictions. Moreover, MuMo proficiently differentiated between high- and low-risk groups in terms of PFS (log-rank test, *P* = 0.0079; Fig. 4f) and OS (log-rank test, *P* = 0.0042; Fig. 4i), which are essential for patient prognosis and treatment planning. This impressive performance highlights the critical role of MuMo in adapting to a relatively novel treatment regimen, such as anti-HER2 combined immunotherapy, where clinician experience and historical data may be limited. The capability of advanced deep learning models, such as MuMo, to extract meaningful insights from various cohorts demonstrates their potential for broad applications in emerging treatment scenarios.

### MuMo’s consistent stability in treatment response prediction

To ascertain the reliability and consistency of MuMo in predicting treatment responses, we conducted a confirmatory experiment using 2000 bootstrap replicates, a statistical method to estimate the sampling distribution, for both the anti-HER2 and anti-HER2 combined immunotherapy cohorts. We calculated the AUC scores for each replicate, and these scores were visually represented using box plots, which effectively illustrated the distribution and variability of the scores. The results demonstrated that MuMo exhibited a small performance variability, which is a key indicator of its consistent stability and reliability in predicting treatment responses (Fig. 4j). Furthermore, MuMo displayed significantly lower performance variability than both individual clinicians and their collective decision-making processes in group consultations (Levene’s test, *P* < 0.05; Supplementary Fig. S6). Additionally, we demonstrated MuMo’s stable predictive performance (AUC 0.800 to 0.833; Supplementary Fig. S7) across five sets of randomly varied doctor annotations (Supplementary Table S5). These outcomes highlight MuMo’s capability to provide dependable and stable predictions in treatment response scenarios.

### Ablation studies of multi-modal information fusion in MuMo

The proposed MuMo offers a comprehensive perspective of patients with GC undergoing anti-HER2 therapy, largely because of its specially designed fusion modules for multi-modal information fusion. To assess the contribution of the MuMo fusion modules to multi-modal information, we conducted three ablation studies.

In our first experiment, we analyzed patients with both radiological and pathological data (Fig. 4k and Supplementary Table S6). Our results showed that integrating radiological and pathological data improved the predictive AUC score of the model to 0.750, which was superior to the AUC scores achieved by models relying solely on radiological (0.639) or pathological (0.703) data. The pathology-only model performed notably better than the radiology-only model, which can be attributed to the comprehensive visual insights provided by pathological analysis. As the clinical gold standard for diagnosis, pathology offers an effective portrayal of the tumor immune microenvironment, which is a crucial determinant of treatment response.^28^

Second, we evaluated the effectiveness of MuMo’s specialized inter-modal fusion module, which includes modal-agnostic feature alignment, to integrate disparate information sources (more details in the methods section). The fusion module showed enhanced performance compared with simple combinations of features derived from radiological and pathological data (Fig. 4l and Supplementary Table S7), such as element-wise multiplication (0.577), summation (0.682), and concatenation (0.731). This underscores MuMo’s ability to effectively consolidate inter-modal information. Moreover, when we assessed a MuMo variant that lacked modal-agnostic feature alignment in the latent space, its performance decreased (AUC scores: 0.772) compared to the full MuMo (0.821), demonstrating the advantage of feature alignment across different modalities in a unified embedding space, as supported by the relevant literature.^10,11^

Finally, we found that incorporating clinical reports led to a promising increase in the AUC score (0.703–0.769; Fig. 4m and Supplementary Table S8). Furthermore, including detailed patient information improved the performance of model (increasing the AUC scores: 0.769–0.821). These findings suggest that comprehensive data from clinical reports and patient-specific information significantly enhanced MuMo’s predictive capabilities.

### MuMo’s interpretability with clinical insights

**Fig. 5:**
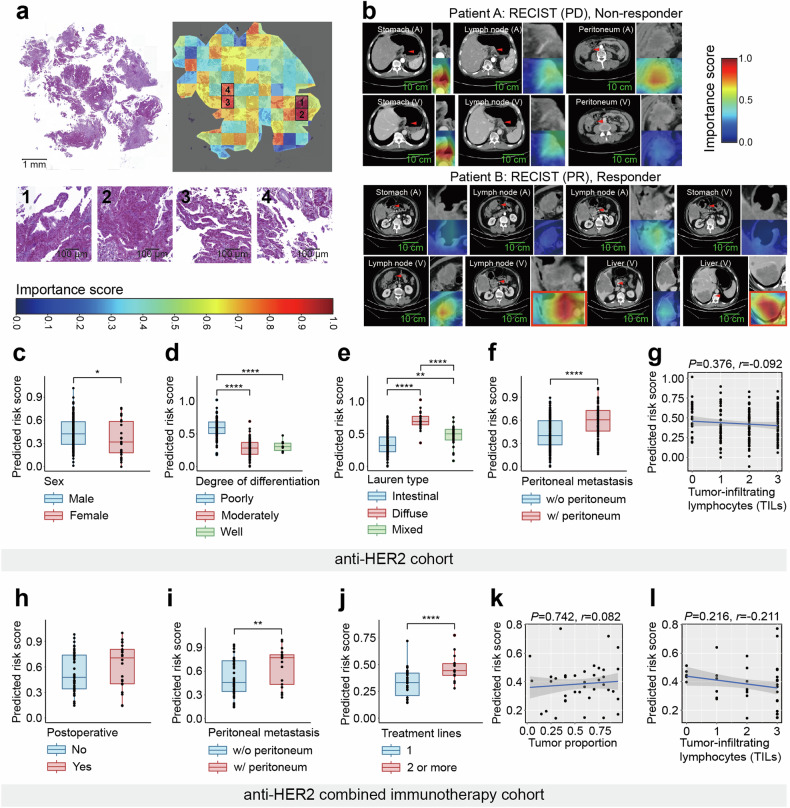
Interpretability analysis of the Multi-Modal Model (MuMo). **a** Visualization of the importance scores of ‘bags’ on pathological whole-slide images. Darker red regions signify a higher contribution to the response prediction, whereas darker blue regions suggest a diminished influence. The second row shows the four most important bags on the slide image. **b** Visualization of attention maps on radiological lesion images using the Grad-CAM algorithm. Darker red regions signify heightened attention from MuMo, whereas darker blue regions denote reduced attention. The red bounding box emphasizes MuMo’s predominant focus on lymph node and liver tumors in this responder. **c**–**g** Evaluation of predicted risk scores across various clinical information subgroups in the anti-HER2 cohort. **h**–**l** Evaluation of predicted risk scores across various clinical information subgroups in the anti-HER2 combined immunotherapy cohort

We validated the alignment between MuMo predictions and established clinical knowledge, focusing on two key perspectives: image-focused regions for visual qualitative analysis and clinical information weights for quantitative analysis. In the pathological whole-slide images, we used regional important scores that quantified the model’s focus on specific areas to highlight where the model concentrated its predictions. Notably, these focus areas correlated intuitively with HER2 (3+) expression regions, characterized by a high tumor-to-stroma ratio (over 50%), well-differentiated tumor glands, abundant tumor-associated immune cell infiltration, and significant desmoplastic stroma surrounding tumor cells, suggesting that MuMo effectively deduces vital tumor information for predictions (Fig. 5a and Supplementary Fig. S8). In radiological CT scans, we found that MuMo was primarily concentrated in regions harboring lesions, as identified through gradient-weighted class activation mapping (Grad-CAM),^29^ aligning with key areas of clinical concern in cancer diagnosis and treatment (Fig. 5b and Supplementary Fig. S9).

**Fig. 6:**
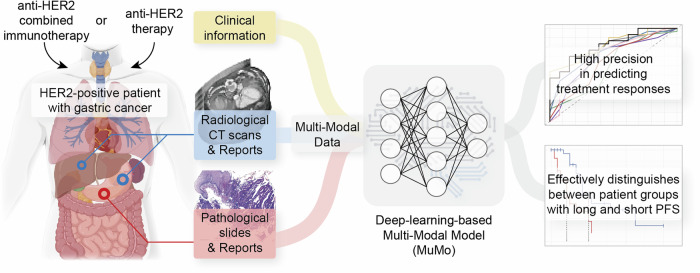
Comprehensive overview of the multi-modal data analysis on HER2-positive patient with GC. This figure illustrates the step-by-step workflow of our research approach, from data collection through to the analysis techniques used. Key results are highlighted, demonstrating the highly prediction accuracy of treatment response of MuMo in HER2-positive patient with GC

Subsequently, we evaluated the risk scores predicted by MuMo across various clinical information subgroups, including patient information, radiologically structured clinical reports, and pathologically structured clinical reports (Fig. 5c–l and Supplementary Fig. S10). We observed that in the anti-HER2 cohort, sex, degree of differentiation, Lauren type, and peritoneal metastasis were identified as key decision variables by the MuMo predicted risk scores. Among these, male patients were predicted to have higher risk scores compared to female patients (Mann–Whitney U test, *P* = 0.041; Fig. 5c). For patients with poorly differentiated tumors (Fig. 5d), MuMo assessed their risk probability as significantly higher compared to those with moderately differentiated tumors (*P* < 0.0001) and well-differentiated tumors (*P* = 0.0003). For Lauren classification (Fig. 5e), MuMo assessed that the diffuse type had the highest risk probabilities, being significantly higher compared to the intestinal type (*P* < 0.0001) and the mixed type (*P* = 0.0038). Conversely, the intestinal type had relatively lower risk, with the mixed type falling between the two in GC. Additionally, MuMo recognized patients with peritoneal metastasis as having significantly poorer responses than those without peritoneal metastasis (*P* < 0.0001; Fig. 5f). Furthermore, an increase in TILs was also seen to slightly reduce the predicted risk scores (Pearson correlation coefficient *r* = −0.092), indicating MuMo’s awareness of the relationship between the abundance and activity of TILs and patient outcomes. In the anti-HER2 combined immunotherapy cohort, the presence of peritoneal metastasis also remained a significant decision variable for predicting high risk scores by MuMo (*P* = 0.027; Fig. 5i); additionally, MuMo identified patients undergoing second-line treatment as having poorer responses compared to those undergoing first-line treatment; similarly, MuMo also recognized a negative correlation (*r* = −0.211; Fig. 5l) between the abundance and activity of TILs and predicted risk probabilities, suggesting that a higher abundance of TILs is associated with better treatment responses. These analyses demonstrate that MuMo can extract appropriate knowledge from clinical reports and patient information to make accurately treatment response predictions, and its recognition of these significant decision variables aligns with current clinical findings,^30,31^ confirming MuMo’s reliability and clinical relevance (Fig. 6).

## Discussion

This study demonstrated the accuracy and utility of multi-modal data analysis in predicting the response to anti-HER2 therapy in patients with HER2-positive GC. Our dataset, which is the largest available as confirmed by a thorough literature search, covers various types of medical information, including patient demographics, radiological CT scans containing multiple lesions, pathologic whole-slide images with different HER2 expression levels (0–3+), and structured clinical reports. Our proposed MuMo excels in predicting the response to both anti-HER2 therapy and anti-HER2 combined with immunotherapy in patients with HER2-positive GC. Importantly, the external validation of the predictive capabilities of MuMo in an independent cohort from other medical centers underscores its potential applicability in diverse clinical settings.

The advantage of MuMo lies in its efficient integration capabilities with multi-modal data, compared to the widely studied unimodal models.^18,22,32–34^ It utilizes a comprehensive and rich patient profile to improve the accuracy of treatment response predictions. Recent studies have started to investigate the use of multi-modal data in treatment response prediction for various cancers, including clear cell renal cell carcinoma,^35^ non-small cell lung cancer,^17,36,37^ and hepatocellular carcinoma.^38^ However, there is a notable scarcity of response prediction research on patients with HER2-positive GC and their response to anti-HER2 therapy. Moreover, current approaches mainly rely on simple integration methods, such as concatenation,^39^ aligning and then averaging,^40^ or multivariate machine learning analysis,^35,38,41^ which do not take into account the potential uniqueness and overlap of information across different modalities in medical field. In contrast, MuMo dynamically decouples modal-agnostic and modal-specific features, facilitating a more rational process of multi-modal fusion by aligning modal-agnostic features. Additionally, MuMo uniquely employs a learnable feature approach to address the issue of missing modalities in real-world scenarios, enabling accurate treatment response predictions for patients with partially incomplete data. Furthermore, to realistically validate MuMo’s application in clinical practice, our study includes extensive comparisons between MuMo’s predictions and the evaluations made by six clinicians. This demonstrates the potential of MuMo to assist in clinical decision-making, highlighting its practical utility in supporting physicians.

Regarding clinical applications, the module design and standardized architecture of our model allow easy scaling to accommodate more lesions, modalities, and time points. For instance, by including more lesions in the CT images, the intra-modal fusion module in the feature extraction process can be readily expanded. The model dynamic structure can adapt to increasingly complex treatment response prediction tasks, such as incorporating patient information from multiple time points. Therefore, the standardized framework allowed us to form a novel continual-learning paradigm that incorporated the model into the entire medical imaging and reporting workflow. Once the new information (images or text) is generated, our model can be trained without waiting for the complete collection of patient information. This is particularly important for providing personalized treatment, as the model can immediately consider the latest diagnostic information, treatment response, and changes in patient status to enable more accurate treatment predictions and improve patient management. For patients undergoing treatment, such modality-wise analysis can quickly identify whether they are unlikely to respond positively to the current treatment regimen, facilitating the prompt adjustment of therapeutic plans. For patients requiring chronic disease management, such meticulous optimization can also capture subtle health changes, providing physicians with real-time feedback on potential complications or signs of disease recurrence.

Another major potential of the MuMo is its ability to integrate more modalities in the future, which is key to realizing personalized treatment strategies. Our model consolidated diverse data sources, including radiological CT, pathological images, and clinical reports, and can incorporate even more modalities in the future, such as cancer biomarkers, gene expression, and lifestyle and health history information. Adding such data may significantly improve the model’s accuracy in predicting treatment response while also helping physicians formulate more targeted treatment plans. For instance, by integrating gene expression data, we can gain deeper insight into patient’s pharmacogenomics, thereby optimizing drug selection and dosage adjustments.

The flexibility of the MuMo makes it an ideal platform for interdisciplinary collaboration, facilitating knowledge fusion between bioinformaticians, clinicians, and data scientists. Through such collaborations, MuMo can continuously assimilate the latest research discoveries and clinical feedback to iteratively update and refine its algorithms. Additionally, the MuMo framework is highly extensible and can be rapidly expanded to other cancer types and diagnostic markers. By undergoing targeted retraining with specific cohorts, MuMo can swiftly adapt to different cancers such as breast cancer or to other clinically relevant immunohistochemical (IHC) markers like ER, PR, and EGFR. This adaptability not only enhances the model’s utility across various oncological applications but also supports a more comprehensive approach to personalized medicine. Furthermore, we hope to explore the use of data collected from wearable devices and remote monitoring tools, such as patient activity levels and physiological responses, which can provide the model with comprehensive health information for more accurate personalized treatment.

Although our preliminary findings using the MuMo are promising, we must acknowledge its limitations. First, although our dataset was collected from multiple medical centers and focused primarily on patients with HER2-positive GC, expansion is possible. Secondly, despite performing accurate treatment response predictions, the model still relies on human input for certain sub-tasks, such as requiring experts to annotate bounding boxes around lesions in radiological images and delineate regions with different HER2 expression levels in pathological slides. In future studies, we plan to incorporate automation techniques, including the deployment of large language models and AI agents, to further reduce the need for human input and move closer to fully autonomous end-to-end treatment response prediction systems.

Summarily, MuMo represents a promising strategy for leveraging AI capabilities to improve response-predictive accuracy in patients with GC receiving anti-HER2 or anti-HER2 combined immunotherapy. By employing a comprehensive, multi-modal dataset, we are making significant strides toward realizing personalized treatment strategies. The model is an impressive testament to the potential integration of diverse modalities and AI, highlighting an exciting direction for future oncology research.

## Materials and methods

### Data collection

Our study included patients recruited between January 2007 and January 2023, who were divided into three distinct cohorts: the anti-HER2 cohort, comprising patients who received anti-HER2 therapy; the anti-HER2 combined immunotherapy cohort, comprising patients treated with anti-HER2 and anti-PD-1/PD-L1 ICI; and an external cohort, comprising patients from external hospitals who received anti-HER2 therapy. The study received approval from the Ethics Committees of Peking University Cancer Hospital, Nanfang Hospital, and Peking University Third Hospital (approval numbers 2020KT08, NFEC2017171, and D2021077, respectively). Informed consent was obtained from all participants or their legally authorized representatives. We asked pathologists and radiologists to annotate the medical images and provide structured clinical reports to ensure comprehensive data collection. Epstein-Barr Virus status was ascertained using in situ hybridization, employing probes targeting Epstein-Barr encoded RNA. Mismatch Repair status, a key factor in determining cancer behavior and treatment response, was assessed using IHC analysis to examine the expression levels of DNA mismatch repair proteins, specifically MLH1, MSH2, MSH6, and PMS2, following previously described methods.^42^

### Survival and response metrics

OS is defined as the time from diagnosis until either the death of the patient or the end of the follow-up period, whichever occurred first. PFS refers to the time from the start of treatment to disease progression, recurrence, or death, whichever occurred first. Responders were defined as patients who achieved the response evaluation criteria in solid tumors (RECIST) designation of complete response (CR), partial response (PR), or stable disease (SD) with PFS exceeding the median PFS reported in KEYNOTE-811^4^ (8 months for the anti-HER2 cohort and 10 months for the anti-HER2 combined immunotherapy cohort). Non-responders included those with a RECIST designation of progressive disease (PD) or SD but did not exceed the median PFS reported in KEYNOTE-811. To maintain the integrity of our data and prevent data distortion, patients lost to follow-up (also referred to as censored cases) before reaching the median PFS threshold were excluded from the response prediction analysis. However, they were considered in the survival analysis to provide a more complete overview of patient outcomes.

### Identifying tumor regions in pathological slides

Hematoxylin-Eosin (H&E) and IHC slides are prepared from consecutive tissue sections, typically cut at a thickness of 4 micrometers. This method ensures that the tissue morphology is essentially congruent between the H&E and IHC slides, facilitating precise mapping of HER2 status (as detailed in Supplementary Text S4) onto the H&E images. Based on these carefully aligned slides, experienced pathologists employed an Automated Slide Analysis Platform (ASAP version 1.9, https://computationalpathologygroup.github.io/ASAP/) to identify tumor regions within pathological whole-slide H&E images (Supplementary Text S5). These regions exhibited varying expression levels of HER2, including regions with HER2 = 0, HER2 = 1+, HER2 = 2+, and HER2 = 3+, effectively illustrating the heterogeneity of HER2 expression within the tumor (Supplementary Fig. S11 and Supplementary Table S9). This process requires the pathologist to meticulously outline the contours of each region, effectively marking the boundaries in a point-by-point manner while avoiding necrotic areas and normal glands. Concurrently, the pathologist summarized the information in H&E images to write structure pathological clinical report. During the entire process, a senior pathologist reviewed and affirmed all these results to ensure their precision and adherence to standard guidelines. Based on these annotation results, we standardized the bag-level pathological images through the Reinhard algorithm and white balance processing to unify the color distribution differences across different slides and centers (Supplementary Figs. S12, S13 and Supplementary Text S6).

### Identifying tumor regions in radiological CT scans

For the radiological data, three radiologists employed the ITK-SNAP software (version 3.6.0, http://www.itksnap.org) to identify and annotate primary (GC) and metastatic lesions (liver, lymph nodes, spleen, bone, and soft tissue) within the 3D CT scans. The metastatic lesions were chosen according to the RECIST v1.1 criteria,^43^ where radiologists selected a maximum of two target lesions per organ and a total of no more than five target lesions. Then the radiologists used minimal bounding boxes to encompass the entire lesion as fully as possible. Simultaneously, the radiologists critically assessed the radiological data and wrote a structured radiological clinical report. A senior radiologist reviewed and validated all results throughout this process, ensuring their precision and adherence to standard guidelines. Based on these annotations, we initially computed dynamic windows for various lesion types and centers (Supplementary Fig. S14 and Supplementary Table S10), using them to normalize the corresponding radiological lesion images (Supplementary Fig. S15 and Supplementary Text S6).

### Overall framework of MuMo

We developed a MuMo, a transformer-based model designed to predict treatment responses to anti-HER2 therapy and anti-HER2 combined immunotherapy (Fig. 2a, b). MuMo begins by extracting diverse features (Fig. 2a), including deep image features, omics features, and clinical reports, from different modalities such as pathological whole-slide images and radiological CT scans, using specialized feature extractors (details in subsequent subsections, Supplementary Fig. S16, and Supplementary Text S7). “Deep features” are image features derived from radiological lesion images and pathological word-level images using a deep learning model MnasNet^44^ (detailed in Supplementary Text S7.1). This method effectively identifies complex patterns that are challenging to distinguish manually. “Omics features” consist of a wide range of radiomics features extracted by PyRadiomics library,^45^ including first-order statistics, shape, texture, and higher-order statistical features, providing a comprehensive quantitative analysis of image data. MuMo then utilizes multi-modal fusion modules (Fig. 2b), including intra-modal fusion, inter-modal fusion, and patient information fusion, to effectively integrate information from different modalities, ensuring a comprehensive and precise analysis for response prediction (details in subsequent subsections, Supplementary Fig. S17, and Supplementary Text S8). Specifically, MuMo employs an intra-modal fusion module, that integrates features within the same modality, such as pathological image features, pathological omics features, and pathological clinical reports, to create a comprehensive set of modality-specific data. MuMo uses an inter-modal fusion module that amalgamates multi-modal features from different modalities into a unified feature. Importantly, this module is tailored to address instances of missing modalities by incorporating learnable modality-specific features, thereby ensuring the robustness of the model even with incomplete datasets. Finally, to enhance the precision of the response predictions, the model considers patient-level clinical information through the patient information fusion module and makes response predictions through a multi-layer perceptron with a softmax activation function.

### Feature extraction in MuMo

In our study, we employed three distinct methodologies to extract diverse features, including deep features, omics features, and clinical reports, from available radiological and pathological data (Fig. 2a, Supplementary Fig. S16, and Supplementary Text S7).

In pathology, drawing insights from the prior study^46^ on high-resolution pathological WSIs, we partitioned WSIs into larger segments known as “bags” within the annotated region-of-interest (ROI) areas for more focused analysis. These “bags” were further subdivided into smaller patches, referred to as “words” (Supplementary Fig. S18). Employing MnasNet, a recent lightweight convolutional neural network, enabled efficient conversion of these “words” into word-level deep features. From these, we extracted patient-level deep features using a bottom-up process (Supplementary Fig. S16a). Additionally, we generated pathological omics features from these “bags” using the PyRadiomics library. Pathological image features combine patient-level deep features with pathological omics features. Furthermore, the pathological clinical reports provided by pathologists were mapped into embeddings using a predefined parameterless encoder (Supplementary Table S11).

In radiology, we began by preprocessing the CT scans to construct focused ROI radiological images following the radiologists’ annotations (Supplementary Fig. S19). Subsequently, we employed MnasNet to extract deep features from the ROI radiological images. Additionally, we segmented the ROI radiological images using a pre-trained lesion segmentor (Supplementary Fig. S20) and utilized the PyRadiomics library to derive the radiological omics features. We combined these two types of features to form radiological image features. Mirroring this approach in pathology, we converted the radiological clinical reports provide by radiologists into embeddings using a predefined parameterless encoder (Supplementary Table S12).

### Multi-modal information fusion in MuMo

We developed intra-modal, inter-modal, and patient information fusion modules, each specifically designed to synthesize and integrate different types of data for enhanced treatment response prediction (Fig. 2b, Supplementary Fig. S17, and Supplementary Text S8).

The intra-modal fusion module begins by processing image features and structured clinical reports (Supplementary Fig. S17a). In this module, image features, comprising deep and omics features, are transformed into functional features, including query (*Q*), key (*K*), and value (*V*), through fully connected layers. Subsequently, a cross-attention layer^47^ then merges the information from *V* and *Q*, according to the mutual interaction between *Q* and *K*. Simultaneously, clinical reports are transformed into embeddings using the parameterless encoder (Supplementary Tables S11–S13), forming another key (*K'*) and value (*V'*). A similar cross-attention layer then merges the information from *V'* and *Q*. Finally, these two aggregated features are integrated with the original deep features via element-wise summation, producing an intra-modal fused feature. The outputs from this module for the pathological and radiological data are denoted as *F*_*path*_ and *F*_*rad*_, respectively.

Second, the inter-modal fusion module takes the aggregated features *F*_*path*_ and *F*_*rad*_ and integrates them, operating under three clinical scenarios:

I. All modality data are available (left part of Supplementary Fig. S17b): In cases where both radiological and pathological data are available for a patient, the corresponding features *F*_*path*_ and *F*_*rad*_ first pass through a fully connected layer. These are then divided into modal-specific (*F*_*path_s*_ and *F*_*rad_s*_) and modal-agnostic (*F*_*path_a*_ and *F*_*rad_a*_) features using dedicated functional layers. Subsequently, an alignment algorithm for modal-agnostic features aligns these features from different modalities via contrastive learning^48^ (see details in Supplementary Text S9.3). An element-wise mean operation computes the averaged modal-agnostic feature. The two modal-specific features and the averaged modal-agnostic feature are then concatenated to form an inter-modal fused feature.
II. Missing radiological data (middle part of Supplementary Fig. S17b): In this scenario, the radiological aggregated feature *F*_*rad*_ is absent. As a substitute, a learnable radiological feature ${F}_{{rad}}^{{\prime} }$, with the same dimensions as *F*_*rad*_, is used as a placeholder. The processing of pathological features *F*_*path*_ remains the same as that in scenario I. Only one modal-agnostic feature from the pathological data is generated, serving as the averaged modal-agnostic feature. The pathological modal-specific, averaged modal-agnostic, and learnable pathological features are concatenated to generate the inter-modal fused feature.
III. Missing pathological data (right part of Supplementary Fig. S17b): This scenario mirrors scenario II but with the use of a learnable pathological feature ${F}_{{path}}^{{\prime} }$ as a placeholder. The radiological modal-specific and averaged modal-agnostic features are concatenated with a learnable pathological feature to form the inter-modal fused feature.

The output feature of the inter-modal fusion, denoted as *F*_*inter*_, served as the input for the patient information fusion module (Supplementary Fig. S17c). *F*_*inter*_ is first mapped onto a query ($Q^{\prime\prime}$) via a fully connected layer. Patient information is initially encoded into embeddings using a parameterless encoder (Supplementary Table S13) and subsequently transformed into a key (*K''*) and value (*V''*) through fully connected layers. A cross-attention layer integrates patient information from *F*_*inter*_, culminating in patient-level features for response prediction.

### Experimental design

For the anti-HER2 cohort with 271 patients, collected from Peking University Cancer Hospital (PKCancer), we randomly split the cohort into a training set of 215 patients for deep learning model training and a validation set of 56 patients for hyperparameter optimization. The specifics of the training configurations are elaborated on in Supplementary Text S6 and S9. Upon completion of the training phase, we averaged the weights of the top seven best-performing models to create our final trained model. This model was subsequently used to further analyze the results (Fig. 2c). To test the generalizability of our model, we compiled an independent test set of 39 individuals from external hospitals, all of whom had received anti-HER2 therapy. The experimental setup for the anti-HER2 combined immunotherapy cohort, also sourced from the Peking University Cancer Hospital, mirrored that of the anti-HER2 cohort. This cohort was partitioned into a training set of 89 patients and a validation set of 30 patients. Analysis of the results was performed for this validation set.

### Statistics and reproducibility

Sample sizes were determined based on the availability of suitable patient data that met the inclusion criteria. No statistical method was used to determine the sample size. To our knowledge, our collected dataset is the most comprehensive to date, covering multiple modalities for patients with GC receiving anti-HER2 therapy or anti-HER2 combined immunotherapy. The different distributions of data characteristics between responders and non-responders were evaluated using the two-sided Mann–Whitney U test for two continuous variables (number of metastatic lymph nodes, tumor proportion, and tumor-infiltrating lymphocytes) and the chi-square test for the remaining categorical variables. Survival functions were estimated using the Kaplan–Meier method, and survival distributions across groups were compared using the log-rank (Mantel-Cox) test. We used Levene’s test to assess consistent stability in treatment response prediction. The discriminative performance of the model was evaluated using the ROC-AUC. For statistical analyses, we used R (version 4.1.3) for survival functions, distributions, and stability assessments and Python (version 3.7.10) for model evaluation. We established a *P*-value threshold of < 0.05 to denote statistical significance in this study.

To ensure reproducibility, we have detailed our methodology in the Supplementary Materials, which covers feature extraction, fusion module operations, loss function definitions, experimental specifics, and evaluation metrics (Supplementary Figs. S16–S20, Supplementary Tables S10–S15, and Supplementary Texts S1–S3 and S6–S9). We also meticulously detailed our data management protocols, including data collection, annotation, and processing (Supplementary Figs. S1–S15 and Supplementary Tables S1–S9 and Supplementary Texts S4–S5). These procedures adhered to good clinical practice and data privacy regulations.

### Supplementary information

Supplementary information

Source code

### Source data

Source data

## Data Availability

All data referenced in this study are available within the article and on GitHub at https://github.com/czifan/MuMo. Due to policy constraints, the raw radiological and pathological data should be obtained by submitting a reasonable request to the corresponding author.

## Code availability

The source code of the proposed Multi-Modal Model (MuMo) is available on GitHub at https://github.com/czifan/MuMo. All R packages employed in this study can be found on CRAN (https://cran.r-project.org/web/packages/available_packages_by_name.html) or Bioconductor (https://www.bioconductor.org/).
