# Supplementary material for: Predicting gastric cancer response to anti-HER2 therapy or anti-HER2 combined immunotherapy based on multi-modal data
Source: Signal Transduct Target Ther. 2024 Aug 26;9:222. doi: 10.1038/s41392-024-01932-y (PMC11345439; doi:10.1038/s41392-024-01932-y)
Supplement: Supplementary file 1 — Supplementary information [file 41392_2024_1932_MOESM1_ESM.docx]

Supplementary Materials for

Predicting gastric cancer response to anti-HER2 therapy or anti-HER2 combined immunotherapy based on multi-modal data

Zifan Chen, Yang Chen, Yu Sun, Lei Tang, Li Zhang, Yajie Hu, Meng He, Zhiwei Li, Siyuan Cheng, Jiajia Yuan, Zhenghang Wang, Yakun Wang, Jie Zhao, Jifang Gong, Liying Zhao, Baoshan Cao, Guoxin Li, Xiaotian Zhang, Bin Dong, Lin Shen

Correspondence to: [zhangxiaotianmed@163.com](mailto:zhangxiaotianmed@163.com); [dongbin@math.pku.edu.cn](mailto:dongbin@math.pku.edu.cn); [shenlin@bjmu.edu.cn](mailto:shenlin@bjmu.edu.cn).

**This PDF file includes:**

Supplementary Texts S1 to S9

Figures. S1 to S20

Tables S1 to S15

**Other Supplementary Materials for this manuscript include the following:**

Code S1 [Code.pdf]

Data S1 [Data.xlsx]

Text S1. External validation on TCGA datasets.

To demonstrate the flexibility of our proposed MuMo framework, we conducted experiments based on the publicly available, TCGA-STAD (<https://portal.gdc.cancer.gov/v1/projects/TCGA-STAD>) and TCGA-BRCA (<https://portal.gdc.cancer.gov/v1/projects/TCGA-BRCA>), which focus on gastric adenocarcinoma and invasive breast carcinoma, respectively. This supplementary section details the methodology encompassing data processing, model structure, experimental details, and preliminary results.

*Data processing:* We obtained H&E-stained WSIs and patient information from NIH national cancer institute GDC data portal (https://portal.gdc.cancer.gov/v1/) and radiological image data from NIH national cancer institute CIP cancer imaging program (<https://www.cancerimagingarchive.net/collection/tcga-stad/>). For the pathology WSIs, we applied a threshold segmentation algorithm (with a threshold value of 200 in gray space) to binarize the slide images, isolating the foreground. Then we cropped bags from the original high-resolution WSI at $2048\times2048$ pixels within the foreground. Subsequent handling of pathology slides and extraction of radiomics features at the bag level were consistent with the original MuMo processes. For the radiology data, we only utilized CT scans in TCGA-STAD due to the varied sequence quality available for TCGA-BRCA. An in-house developed gastric cancer auto-segmentation model^1^ was used to localize and segment the primary gastric lesions in 3D CT scans. Subsequent handling of radiological images and extraction of radiomics features from primary lesions followed existing MuMo protocols. For patient information, we extracted several clinical features (nine for TCGA-STAD, including age, sex, degree of differentiation, pathologic M, pathologic N, pathologic T, the lymph node examined count, and the number of lymph nodes positive by he; eight for TCGA-BRCA, except no differentiation, consistent with TCGA-STAD) and encoded them into embeddings using a pre-defined parameterless encoder (Supplementary Tables S14–S15). Due to lacking treatment efficacy data from TCGA, we used the median overall survival (353 days for TCGA-STAD; 1223 days for TCGA-BRCA) to categorize patients into two groups: label=0 for those with OS below the median and who were not censored, and label=1 for those with OS above the median. We randomly split the datasets into training, validation, and test sets with a ratio of 70:15:15. For TCGA-STAD, this resulted in a training set of 290 patients, a validation set of 62 patients, and a test set of 63 patients. Supplementary Figure S4a shows the distribution of proportions for pathological and radiological image data across different sets. For TCGA-BRCA, we obtained a training set of 743 patients, a validation set of 159 patients, and a test set of 160 patients.

*HER2-positive identification:* To identify HER2-positive patients, we utilized TCGA Genotyping data. Specifically, we analyzed the HER2 gene region on chromosome 17 (chr17: 37844198 to 37886679) across patient samples. We calculated the copy number for the HER2 region in each sample and classified the top 20% of patients with the highest HER2 copy numbers as HER2-positive. Using this method, we identified a total of 66 HER2-positive patients from the TCGA-BRCA validation and test sets.

*Model structure:* The overall structure of MuMo is designed for flexible expansion, without altering the core architecture of MuMo. Due to the temporary unavailability of radiological and pathological clinical reports, we omitted the modules that integrate these clinical reports (corresponding to the right side of Supplementary Figure S17a).

*Experimental details:* We did not adjust any hyperparameters, instead, training was conducted using the hyperparameters from MuMo’s original deployment in the anti-HER2 cohort. Due to the absence of physician-sketched ROI annotations in TCGA data, we removed the auxiliary task loss introduced in Supplementary Text S7.4. All remaining experimental details are consistent with the original MuMo. We called this MuMo trained on TCGA datasets as MuMo-TCGA.

*Preliminary results:* The performance of MuMo-TCGA on the TCGA-STAD and TCGA-BRCA datasets is reported in Supplementary Figures S3–S4. Without hyperparameter tuning specifically for the TCGA datasets, our MuMo model demonstrated respectable performance. It achieved an AUC score of 0.698 on the TCGA-STAD validation set and 0.686 on the TCGA-STAD test set (Supplementary Figures S4b–S4c). Additionally, the model achieved an AUC score of 0.706 on the TCGA-BRCA validation set, 0.630 on the TCGA-BRCA test set, and 0.732 on TCGA-BRCA HER2-positive patients (Supplementary Figures S3a–S3c). The performance gap compared to the original MuMo in HER2-positive GC is influenced by two main factors:

1) the absence of physician-sketched ROIs and specialized clinical reports in TCGA, reducing the professional prior knowledge integrated into MuMo and resulting in the unavailability of the auxiliary loss described in Supplementary Text S7.4;

2) in the TCGA datasets, pathology data predominantly outweighs radiological data (specific proportions detailed in Supplementary Figure S4a; 88.92% of the data in TCGA-STAD is solely pathology; all data in TCGA-BRCA exclusively uses pathology), with MuMo-TCGA’s performance being comparable to that shown in the pathology-only ablation studies of our original MuMo (0.698 vs. 0.703; Figure 4k).

This external validation experiments on TCGA demonstrate MuMo’s flexibility, scalability, and reusability, and reveals that incorporating a more comprehensive set of multi-modal data could potentially enhance MuMo’s performance further.

Text S2. Additional validation of MuMo for rapid expansion of molecular pathology factors.

The data we collected has substantial missing information or a strong bias towards a certain expression for molecular pathology factors (as shown in Table 1), with approximately half of the PD-L1/MMR/EBV data missing. Additionally, EBV is almost entirely negative, and MMR is predominantly pMMR. Consequently, our original MuMo model was not trained and modeled to include these molecular pathology factors. However, MuMo’s design is flexible, allowing us to quickly incorporate new knowledge, such as molecular pathology factors, by treating them as new elements of patient information.

*Experimental details:* We conducted an additional validation on the anti-HER2 validation set to test MuMo’s capability to rapidly integrate new molecular pathology factors (without retraining MuMo). First, we encoded PD-L1, MMR, and EBV into one-hot embeddings similar to the method described in Supplementary Table S13. Each factor embedding was then mapped to the required feature dimension of the patient information fusion module using randomly initialized linear layers. These factors were considered as additional dimensions in the patient information feature sequence. For example, if the original patient information features were $N\times C$, after adding PD-L1, MMR, and EBV, they became $\left( N+3 \right)\times C$. The modified features were then used directly for inference.

*Preliminary results:* The ROC curves for this straightforward extension experiment are shown in Supplementary Figure S5. It can be observed that the addition of each factor results in slight performance improvements for the original MuMo. Specifically, the inclusion of PD-L1 increased MuMo’s AUC score from 0.821 to 0.831 in the anti-HER2 validation cohort and from 0.914 to 0.936 in the anti-HER2 combined immunotherapy cohort. Although EBV and MMR factors are largely homogeneous, it is important to note that we use an attention mechanism for feature interaction and integration between patient information and inter-modal fusion features (introduced in Supplementary Text S8.3). This ensures that even factors with the same expression can interact with other individual-specific information from inter-modal fusion features to produce personalized outputs. These preliminary results demonstrate MuMo’s flexibility in accommodating new knowledge and highlights the importance of IHC factors in efficacy assessment tasks. In future work, we plan to collect more comprehensive and complete molecular pathology data to enhance MuMo’s overall capabilities.

Text S3. Evaluation metrics.

This study utilizes two primary evaluation metrics: the Receiver Operating Characteristic-Area Under the Curve (ROC-AUC) for assessing treatment response prediction and the Kaplan-Meier curves for survival analysis.

*ROC-AUC:* The ROC-AUC curve measures the effectiveness of our model and doctors' annotations for treatment response prediction. This curve captures the model’s ability to differentiate between responders and non-responders. The ROC-AUC curve is a graph illustrating the true positive rate (TPR) against the false positive rate (FPR) across a variety of thresholds for classifying positive and negative samples. In essence, the ROC curve assesses the tradeoff between sensitivity (TPR) and specificity (1-FPR) of a binary classifier. The AUC is a summarizing statistic that measures the model's overall performance. AUC values range from 0 to 1, with 0.5 representing a model with no predictive power, and 1 representing a perfect model. Generally, a higher AUC signifies superior model performance. The 95% confidence interval for the AUC is obtained using the “ci.auc” method in R software, which by default employs the DeLong method for calculation. Additionally, in Figure 4k–m, we have employed the bootstrapping method with 100 repetitions to plot the performance of multiple sampled data points, thereby accurately conveying the variability and individual data points.

*Kaplan-Meier analysis:* The Kaplan-Meier (KM) curve assesses whether the model could categorize patients into low-risk and high-risk groups based on their predicted risk scores. The grouping threshold is determined by the Youden Index of the corresponding ROC-AUC curves. The KM curve, a graphical representation of survival analysis, plots survival probability over time. It is commonly employed in medical research and other fields to examine time-to-event data, such as the time to disease recurrence or death. The KM curve estimates the survival probability at each point in time based on the observed data, and it depicts this as a stepwise function. The curve shows the proportion of individuals surviving past a given time point and the number of individuals at risk at that moment. It is often used to compare survival rates among two or more groups and to identify factors potentially impacting survival.

Text S4. HER2 assessment method.

All surgical/endoscopic biopsy formalin-fixed paraffin-embedded (FFPE) gastric tumor samples were initially tested for HER2 using immunohistochemistry (IHC). The IHC test was performed on the automated Bench Mark Ultra system using the VENTANA 4B5 antibody (Roche, Tucson, AZ, USA). Specimens were cut into 4µm slides, with antigen retrieval facilitated by a pH 8.4 EDTA solution incubated at 95°C for 36 minutes. Positive and negative controls were included in every staining run. For surgical sample evaluation, a 10% cut-off value was established, whereas a single cluster of at least 5 positive cells was required for endoscopic biopsy samples. HER2 membrane staining was evaluated as 0, 1+, 2+, or 3+ based on the 2018 ASCO/CAP guidelines,^2^ with an IHC score of 3+ defining HER2 positivity and scores of 0 or 1+ indicating negativity. Samples scored at 2+ underwent further testing using fluorescence in situ hybridization (FISH) to determine their final HER2 status.

HER2 FISH testing was conducted on FFPE samples using the Path Vysion HER2 DNA Probe Kit (Abbott Molecular, Abbott Park, Illinois) as per the manufacturer’s instructions. Experienced pathologists blindly measured the HER2 and CEP17 copy numbers in 20 tumor cells. A HER2/CEP17 ratio of ≥2.0 was considered positive, a ratio of <2.0 was considered negative, and samples with a ratio between 1.8 and 2.2 were retested on an additional 20 nuclei, making a total count for 40 nuclei to determine the final status.

We believe that this addition will provide readers with a comprehensive understanding of the methods used to assess HER2 status in our study, thus enhancing the manuscript’s clarity and depth. This updated information has been included in the manuscript from lines 611–613 on page 24.

Text S5. Methodology for whole-slide imaging and tissue analysis.

The digitized whole-slide images (WSIs) were acquired at Beijing Cancer Hospital using 3DHISTECH slide converter 2.3 viewed at 400× magnification. The biopsy and surgical tissues ware sequentially sliced to produce HE and HER2 slides, so the tissue morphology of HE and HER2 were basically consistent. Average area of surgical specimen of gastric cancer is approximately 200 mm^2^ and for biopsy specimen is 12 mm^2^.

Text S6. Experimental details.

*Normalizing pathological images:* Pathological whole slide images (WSIs) often exhibit batch effects due to variations in staining procedures and conditions across different batches, times, and centers. These variations can manifest as domain shifts in computer vision. We identify two primary types of domain shifts in pathological WSIs:

1) Variations in staining intensity: Differences in the application and duration of staining procedures can lead to variations in color intensity. These differences can be visualized in Supplementary Figure S12a.

2) Yellowing of slide images: Some slide images may exhibit yellowing due to storage conditions or imaging issues, as shown in Supplementary Figure S13.

To address these challenges, we preprocess the pathological WSIs at bag level before model training. For the first type of domain shift, we employ the Reinhard color normalization algorithm, tailored to our specific dataset. The target image for normalization is a reference slide selected by our pathologists, ensuring consistency in staining appearance across different images. Our modified Reinhard algorithm specifically computes color normalization based on foreground elements only, filtering out the background (lighter areas) using a thresholding method (with a threshold value of 200 in gray space). The visual results after enhanced Reinhard normalization are displayed in Supplementary Figure S12b. For the second type of problem, we filtered yellowing slide images during the pathologist review and annotation stage, manually removing severely yellowed images and selecting mildly yellowed images. Subsequently, we applied white balancing algorithm to adjust the color proportions to ensure the background area is white. The visual results after white balancing are shown in Supplementary Figure S13.

*Normalizing radiological images:* Radiological CT scans are subject to imaging variations due to several external factors, such as differences in equipment models and the dosages of contrast agents used. These variations present a concrete example of domain shift issue in the field of computer vision. For our dataset, we have collected data from three medical centers: PKCancer hospital, Peking University Third Hospital, and Nanfang Hospital. To address the issue of domain shifts, we first determined the dynamic window levels and widths for various lesion types from different medical centers (Supplementary Table S10) during radiologist review and annotation stage according to their HU distribution (Supplementary Figure S14). Subsequently, we applied these dynamic window levels and widths to normalize radiological ROI images. Supplementary Figure S15 visualize the comparative results of radiological images with and without normalization.

*Implementation details:* We developed our model using PyTorch^3^ (version 1.10.2) equipped with CUDA 11.0. In radiology, we resized each cropped ROI lesion image into 112$\times112$ pixels for input. In pathology, we cropped bags from the original high-resolution whole-slide image at $2048\times2048$ pixels and downscaled them into $448\times448$ pixels for input. Each bag was further divided into words with $64\times64$ pixels. We trained our model separately in the anti-HER2 cohort and anti-HER2 combined immunotherapy cohort. For each model, we initially pre-trained it using a configuration of $T=360$ epochs, a batch size of 16, and 4 workers. In the pre-training phase, we differentiated responders and non-responders based on both overall survival (OS) and RECIST criteria. Our rationale was that OS, being a more objective fact, would be advantageous for the initial training of the model. Subsequently, we conducted fine-tuning for an additional 100 epochs. This stage involved categorizing responders and non-responders using RECIST and progression-free survival (PFS), as outlined in our paper. During this fine-tuning phase, we judiciously reduced the learning rate to 5e-6 for all parameters except the predictor. We applied the Stochastic Gradient Descent^4^ (SGD) optimizer to optimize the overall loss function, initiating with a learning rate of 1e-3, a weight decay of 1e-4, and a momentum of 0.9. During training, we used the multi-step learning rate decay strategy to decrease the learning rate at the 120-th and 240-th epoch by a decay rate of 0.5. Moreover, to mitigate overfitting and enhance the model's generalizability, five bags, and four lesions were randomly sampled with the replacement for each patient at each epoch during training, simulating image-level dropout. Besides, for both pathological and radiological images, we apply various data augmentation techniques including random flipping, rotation, scaling, cropping, and adjustments in color and contrast.

*Statistical analysis details:* Furthermore, all statistical analyses were conducted using R (version 4.1.3) and Python (version 3.7.10). Categorical variables were analyzed using either the chi-squared test or Fisher's exact test, whereas continuous variables were analyzed using the Mann-Whitney U test. The Levene test was employed to evaluate the performance stability of different methods. In our study, a *P*-value less than 0.05 was considered to denote statistical significance.

Text S7. Details of the feature extraction process within MuMo.

Supplementary Figure S16 provides a detailed depiction of the image feature extraction process. In the following, we outline the feature extraction pipeline for radiology (Supplementary Figure S16a) and pathology (Supplementary Figure S16b). Thereafter, we delve into the particulars of the various modules incorporated in feature extraction, including MnasNet^5^, the correlation module, the importance aggregator, and the pre-trained lesion segmenter.

In pathology, as illustrated in Supplementary Figure S16b, we initially subdivided the high-resolution whole-slice pathological image into large patches, known as bags, according to annotated ROI regions, which were further divided into smaller patches, known as words. All words were converted into deep features using MnasNet. We then utilized a correlation module followed by an importance aggregator to detect correlations between words within each bag and amalgamate them into a unified feature per bag. Likewise, we employed another correlation module and importance aggregator to execute analogous operations between bags and generate a unified feature for each patient. This bottom-to-up process of feature extraction formed the basis for deep feature extraction in pathology, with an MLP utilized to generate pathological deep features. Furthermore, we extracted pathological omics features from bags using the PyRadiomics library. In combination, these pathological deep features and pathological omics features constituted pathological image features. Besides, we also asked pathologists to review the pathological data and wrote structured pathological structured clinical reports. These reports would be converted into embeddings via the pre-defined parameterless encoder, see Supplementary Table S11 for details.

In radiology, as depicted in Supplementary Figure S16a, we employed a deep learning feature extractor known as MnasNet^5^ to derive deep features from preprocessed region-of-interest (ROI) radiological images. Subsequently, we deployed a correlation module to discern intercorrelations between objects (lesions in this case) and generate enhanced features. We then applied the importance aggregator to assign importance scores for each lesion and amalgamate multi-lesion features into a unified feature for each patient. Lastly, we used a multilayer perceptron (MLP) to produce radiological deep features. Concurrently, we segmented preprocessed ROI radiological images using a pre-trained lesion segmenter (Supplementary Text S7.4) and employed the PyRadiomics library^6^ to extract radiological omics features. In combination, these radiological deep features and radiological omics features constituted the radiological image features. Besides, the structured radiological structured clinical reports provided by radiologists would be converted into embeddings via the pre-defined parameterless encoder, see Supplementary Table S12 for details.

Text S7.1. MnasNet.

Given the volume of data in this study and potential future applications, we opted for MnasNet^5^ as our deep learning backbone in both pathological and radiological images. MnasNet, a well-regarded automated mobile architecture search network, strikes a fitting balance between performance and efficiency. The two most important aspects of MnasNet were 1) defining the search space, and 2) crafting a search algorithm to find architectures. Regarding the former, MnasNet proposes a novel factorized hierarchical search space that factorizes a CNN model into unique blocks, then searches for operations and connections per block independently. This design promotes diverse layer architectures in different blocks, enhancing the network’s variety. Each block’s search space consists of convolutional ops, convolutional kernel size, squeeze-and-excitation, skip ops, output filter size, and the number of layers within the block. For the latter aspect, assume the model is $m$, and let $ACC(m)$ and $LAT(m)$ denote $m$’s accuracy on the target task and inference latency on the target mobile platform, respectively. The overall search objective is then defined as,

$$\mathrm{maximize}_{m}\mathrm{ACC}\left( m \right),$$

$$s.t. LAT\left( m \right)\leq T,$$

where $T$ is the target latency. To approximate Pareto optimal solutions to this problem, MnasNet employs a customized weighted product method, with the optimization goal defined as,

$$\mathrm{maximize}_{m}\mathrm{ACC}\left( m \right)\times\left[ \frac{\mathrm{LAT}\left( m \right)}{T} \right]^{w},$$

where $w$ is the weight factor defined as,

$$w=\left\{ \begin{aligned} \alpha if LAT(m)\leq T \\ \beta\mathrm{otherwise} \end{aligned}. \right.$$

These constants, $\alpha$ and $\beta$, are application-specific and can be varied to build different network architectures. Lastly, a reinforcement learning (RL) approach is deployed to find Pareto optimal solutions for the search problem.

More specifically, we selected MnasNet with $\alpha=1.0$ as the deep learning backbone in this study to balance performance and efficiency. This architecture has 4,383,312 parameters and requires 0.31 GFLOPs. We adopted the implementation provided by Torchvision (<https://pytorch.org/vision/stable/models/mnasnet.html>) and used the pre-trained weights, downloaded from the PyTorch model base (<https://download.pytorch.org/models/mnasnet1.0_top1_73.512-f206786ef8.pth>), in our experiments. This weight was, pre-trained on the ImageNet-1K dataset^7^, had achieved top-1 and top-5 accuracies of 73.56% and 91.51%, respectively. MnasNet was deployed to extract deep features in our proposed MuMo, we substituted its classifier with a fully connected layer of lower dimensionality to map 1,280-dimensional features into 512 dimensions features.

Text S7.2. The correlation module.

In this study, we adopted the self-attention mechanism^8^ as a correlation module to mine relationships between multiple objects and to generate enhanced features. The left part of Supplementary Figure S16c shows the specifics of the correlation module.

Suppose the input to the correlation module consists of a set of features, denoted as $F_{cor}\in\mathbb{R}^{N\times C}$, for $N$ objects. The module first maps this input into Query ($Q$), Key ($K$), and Value ($V)$ using three separate functional layers. We then can derive the correlation between objects for $Q$ and $K$ by applying masked matrix multiplication with a Softmax activation, resulting in a correlation matrix (also known as a heatmap representation). Next, we multiply the correlation matrix by $V$ to yield an enhanced feature incorporating the correlation information between objects. Lastly, the enhanced feature passed through a multi-layer perceptron (MLP) with dropout and a residual path to obtain the final prediction, denoted as $F_{cor}^{'}\in\mathbb{R}^{N\times C}$.

Note that, in pathology, the objects are interpreted as bags or words at different levels of the bottom-to-up feature extraction process, while in radiology, the objects are interpreted as lesions. We set the feature dimension, $C$, to 512, correlating with the features extracted from MnasNet as explicated in Supplementary Text 7.1. To mitigate the number of parameters and avert overfitting, we employed a single head of the self-attention mechanism within the correlation module. Furthermore, we set the dropout ratio to 0.2 in the MLP of the correlation module.

Text S7.3. The importance aggregator.

The correlation module (Supplementary Text 7.2) generated an enhanced feature ($F_{cor}^{'}\in\mathbb{R}^{N\times C})$ for $N$ objects. However, the number of objects can vary from patient to patient. For instance, t different patients may exhibit varying numbers of lesions, and the resolution of pathological images can differ, resulting in varying numbers of bags. As such, it became necessary to aggregate multiple objects’ features into a unified feature before making a treatment response prediction. To address this, we introduced a simple importance aggregator in this study., with specifics detailed in the right part of Supplementary Figure S16c.

The input of this module, $F_{cor}^{'}\in\mathbb{R}^{N\times C}$, derived from the output of the corresponding correlation module, passes through a fully connected (FC) layer ($\varphi: \mathbb{R}^{C}\to\mathbb{R}^{1})$. This layer compresses the $C$-dimensions feature into a single dimension, yielding importance scores ($S_{imp}\in\mathbb{R}^{N\times1})$ for each object. The layer $\varphi$ functions without bias and is followed by Softmax activation and a dropout layer with a drop ratio of 0.2. We then utilized these importance scores to aggregate multiple objects’ features, as defined by,

$$F_{agg}=\sum_{i=1}^{N} F_{cor,i}^{'}\times S_{imp,i},$$

$$F_{agg}^{'}=\mathrm{FNN}\left( F_{agg} \right)+F_{agg}.$$

Here, $\mathrm{FNN}$ represents the Feed Forward Network, which consists of two FC layers. The first FC layer is followed by ReLU activation and a dropout layer with a 0.2 drop ratio, mapping the features from $C$ to $2C$ dimensions. The second FC layer, followed by a dropout layer with a 0.2 drop ratio, maps the features from $2C$ back to $C$ dimensions. Note that the input of $\mathrm{FNN}$ is initially normalized by layer normalization^9^. Furthermore, a residual path is employed to combine the input and the output of $\mathrm{FNN}$, thereby generating a unified aggregated feature $F_{agg}^{'}\in\mathbb{R}^{C}$ based on aggregating multiple objects’ features.

The exact interpretation of the unified aggregated feature depended on its specific application. In pathology, it referred to the feature produced by aggregating the words’ features within each bag or aggregating the bags’ features for each patient. In radiology, the aggregated feature referred to the feature produced by aggregating multiple lesions’ features for each patient.

Text S7.4. The pre-trained lesion segmenter.

In this study, we have pre-trained two UNet models to segment stomach lesions and metastatic lesions, respectively. Initially, we collected 2533 2D images of stomach lesions and 2662 2D images of metastatic lesions. These images were garnered from our previous studies, ensuring no overlap with the current research data. Thereafter, we employed the 2D UNet structure from nnUNet^10^ (<https://github.com/MIC-DKFZ/nnUNet/tree/nnunetv1>) to train two distinct segmentation models for the segmentation of stomach lesions (#1) and metastatic lesions (#2), respectively. The segmentation results of these models are illustrated in Supplementary Figure S20. Lastly, based on the segmentation results in conjunction with the original images, we extracted omics features via the PyRadiomics library.

Text S8. Details of fusion modules within MuMo.

In this study, we devised three fusion modules: the intra-modal fusion module (Supplementary Figure S17a), the inter-modal fusion module (Supplementary Figure S17b), and the patient information module (Supplementary Figure S17c). Initially, the image features and the embeddings encoded from structured clinical reports in pathology or radiology were amalgamated via the intra-modal fusion module. Subsequently, these fused features underwent a secondary fusion process orchestrated by the inter-modal fusion module, specifically tailored to address three different situations prevalent in clinical practice. Lately, the patient’s information was incorporated through the patient information module, leading to a patient-level feature that was subsequently processed through the treatment response predictor to predict a score. The ensuing sections will shed light on the details of these three fusion modules. It is worth noting that the output dimension of all fully connected (FC) layers mentioned hereafter consistently amount to 512. Moreover, the number of heads within all cross-attention layers mentioned hereafter was set to 1, with accompanying dropout layers maintaining a drop ratio of 0.2.

Supplementary Text S8.1. The intra-modal fusion module.

As depicted in Supplementary Figure S17a, the intra-modal fusion module takes image features and the associated structured clinical reports supplied by experts as input. Initially, the image features, formed by omics features and deep features, are passed through a series of functional linear layers to generate distinct functional features: Query ($Q$), Key ($K$), and Value ($V$). Subsequently, a cross-attention layer is employed to consolidate the information from $V$ and $Q$, taking into account their mutual interplay. Simultaneously, the clinical report data is transformed into embeddings utilizing the parameterless encoder (as described in Supplementary Text S7), thereby serving as another Key ($K'$) and Value ($V'$). Analogous to the process applied to the omics features, anothor cross-attention layer is employed to consolidate the information from $V'$ and $Q$. Finally, these two aggregated features, in conjunction with original deep features, are combined in an element-wise sum to generate the intra-modal fused feature. We denote the pathological and radiological aggregated features produced by the intra-modal fusion module as $F_{p}$ and $F_{r}$, respectively.

Supplementary Text S8.2. The inter-modal fusion module.

The aggregated features $F_{p}$ and $F_{r}$ produced by the intra-modal models are served as the inputs for the inter-modal fusion module. As shown in Supplementary Figure S17b, the inter-modal fusion module operates under three different situations in clinical practice:

1. *All Modal Data Available*: Here, both radiological and pathological data for the patient are available, yielding corresponding features $F_{p}$ and $F_{r}$. These features first traverse a fully connected layer before being partitioned into modal-specific ($F_{ps}$ and $F_{rs}$) and modal-agnostic features ($F_{pa}$ and $F_{ra}$) via corresponding functional layers. Subsequently, an alignment algorithm for modal-agnostic features aligns these features from different modalities. This step is carried out through contrastive learning (see details in Supplementary Text S9.3). An element-wise mean operation is then performed to obtain the averaged modal-agnostic feature. Finally, the two modal-specific features and the averaged modal-agnostic feature are concatenated to generate the inter-modal fused feature (see the left part of Supplementary Figure S17b).
2. *Missing Radiological Data*: Under these circumstances, the radiological aggregated feature $F_{r}$ is unavailable due to the absence of radiological data. As a substitute, a learnable radiological feature $F_{r}^{'}$, sharing the same dimensions as $F_{r}$, serves as a placeholder. The operations applied to pathological features remain identical to those in the situation I. Furthermore, only a modal-agnostic feature derived from pathology is generated, which is treated as the averaged modal-agnostic feature. Finally, the pathological modal-specific feature, the averaged modal-agnostic feature, and the learnable pathological feature are concatenated to generate the inter-modal fused feature (see the middle part of Supplementary Figure S17b).
3. *Missing Pathological Data*: This situation mirrors situation II, however, it involves the employment a learnable pathological feature $F_{p}^{'}$ as a placeholder. The radiological modal-specific feature and the averaged modal-agnostic feature are concatenated with the learnable pathological feature to generate the inter-modal fused feature (see the right part of Supplementary Figure S17b).

Note that the learnable radiological or pathological feature was initialized as a zero vector in this study. And we denote the generated inter-modal fusion feature as $F_{inter}$.

Supplementary Text S8.3. The patient information fusion module.

The generated inter-modal fusion feature $F_{inter}$ is the input of the patient information fusion module. Within this module, as depicted in Supplementary Figure S17c, $F_{inter}$ is mapped into the Query ($Q$) via a fully connected layer. And the patient information is initially encoded into embeddings using a parameterless encoder (Supplementary Table S13), and subsequently mapped to the Key ($K$) and Value ($V$) through fully connected layers. Lastly, a cross-attention layer is employed to integrate patient information into $F_{inter}$, generating a unified patient-level feature $F$.

Text S9. The definition of loss functions.

In this study, the samples are denoted as $\left( x_{i}, y_{i}, o_{i}, e_{i} \right)$, where i$=1, 2,\cdots, N$. Here, $x_{i}$ represents the input data, including radiological data, pathological data, radiological structured clinical reports, pathological structured clinical reports, and patient information. $y_{i}$ denotes the ground truth of response, defined as responders and non-responders. $o_{i}$ represents overall survival (OS), while $e_{i}$ serves as the event indicator, with 1 representing death and 0 signifying censoring. Based on this, several loss functions were adopted for training MuMo, including cross-entropy loss, deep survival loss, contrastive loss, and intermediate supervised losses.

Text S9.1. The definition of cross-entropy loss.

The first loss function employed in MuMo training was the cross-entropy (CE) loss, utilized to distinguish patients into responders and non-responders. For this binary classification task, the CE loss function was defined as:

$$l_{ce}=-\sum_{i} \left( y_{i}\log_{2} h_{\theta}\left( x_{i} \right)+(1-y_{i})\log_{2}(1-h_{\theta}\left( x_{i} \right)) \right).$$

Here, $\theta$ denotes the learnable parameters within MuMo, while $h_{\theta}$ signifies the two-dimensional output of MuMo. Specifically, the first dimension relates to non-responders (also known as risk scores), and the second dimension relates to responders (also known as survival scores).

Text S9.2. The definition of deep survival loss.

While the cross-entropy loss function is crucial for distinguishing between responders and non-responders, it does not account for the relative risk among patients. For this study, we also considered the overall survival rank relationship among different patients. Following DeepSurv^11^, we utilized the deep survival loss to guide the model in generating effective risk scores based on overall survival, by computing the negative partial log-likelihood as follows:

$$l_{ds}=-\sum_{i,e_{i}=1} \left( h_{\theta}\left( x_{i} \right)_{0,:}-\log\sum_{j\mathfrak{\in R}\left( o_{i} \right)} e^{h_{\theta}\left( x_{j} \right)_{0,:}} \right),$$

where $\mathfrak{R(}o_{i})$ donates the set of patients whose overall survival exceeds that of the $i$-th patient within one mini-batch. The term $h_{\theta}\left( x_{i} \right)_{0,:}$ refers to the risk scores as predicted by MuMo with parameter $\theta$.

Text S9.3. The definition of contrastive loss.

As elucidated in Supplementary Text S9.2, we used contrastive learning to align inter-modal-agnostic features. Initially, corresponding to the three situations in clinical practice, we introduce three sets: $\Omega$, denoting the availability of all modal data; $\Omega_{p}$, denoting the absence of radiological data; $\Omega_{r}$, denoting the absence pathological data. Subsequently, inspired by SimCLR^12^, we defined a positive pair in our study as the paired modal-agnostic features $F_{pa,i}$ and $F_{ra,i}$ derived from the $i$-th patient, and a negative pair as the modal-agnostic features from different patients across modalities. Consequently, the contrastive loss can be expressed as:

$$l_{cl}=-\frac{1}{N}\sum_{i\in\Omega} \log\frac{\frac{\exp\left( F_{pa,i}F_{ra,i} \right)}{\tau}}{\sum_{k\in\Omega} \left[ k\neq i \right]\left( \frac{\exp\left( F_{pa,i}F_{ra,k} \right)}{\tau}+\frac{\exp\left( F_{pa,k}F_{ra,i} \right)}{\tau} \right)},$$

where $\tau$ is the temperature coefficient, which we consistently set at 1.0 for our study.

Text S9.4. The definition of intermediate supervised loss.

The cross-entropy loss and deep survival loss function as patient-level supervision, which, while potent, remains sparse due to the limitation of the patients count. Conversely, the dataset encompasses thousands of lesions, tens of thousands of bags, and hundreds of thousands of words. To augment the efficiency of MuMo, we employed three intermediate supervised losses to simulate the model’s acquisition of more refined features. In this context, we use $\theta_{pb}$ and $\theta_{pw}$ to denote the parameters for bags or words of the pathological deep feature extractor, respectively. We simply characterize the bottom-to-up feature extraction process in pathology as $f_{\theta_{p}}(x_{p,i})$, where $\theta_{p}=\theta_{pb}\cup\theta_{pw}$, and $x_{p,i}$ represents the $i$-th pathological patch. Besides, $\theta_{r}$ represents the parameters of the radiological deep feature extractor, with the output of this extractor described as $f_{\theta_{r}}\left( x_{r,i} \right)$, where $x_{r,i}$ is the image of the $i$-th lesion.

First, we defined an intermediate supervised loss for acquiring the categorization of lesions in radiology, expressed as:

$$l_{r}=-\sum_{i} y_{r,i}\log_{2} f_{\theta_{r}}\left( x_{r,i} \right),$$

where $y_{r,i}$ refers to the ground truth of the $i$-th lesion, translated into a one-hot embedding from the lesion’s categories, which include the stomach, liver, lymph node, peritoneum, and others.

Second, we introduced an intermediate supervised loss for learning the categorization of bags in pathology, expressed as:

$$l_{pb}=-\sum_{i} y_{pb,i}\log_{2} f_{\theta_{pb}}\left( x_{pb,i} \right),$$

where $y_{pb,i}$ refers to the ground truth of the $i$-th bags, translated into a one-hot embedding from the bag’s categories, which include HER2 0/1, HER2 2, and HER2 3.

Third, we introduced an additional intermediate supervised loss, applied at the word level, to learn the categorization of words in pathology, expressed as:

$$l_{pw}=-\sum_{i} y_{pw,i}\log_{2} f_{\theta_{pw}}\left( x_{pw,i} \right),$$

where $y_{pw,i}$ refers to the ground truth of the $i$-th words.

Note that the categorization of words or bags is determined based on the category associated with the most substantial region within the corresponding image.

Text S9.5. The overall loss function.

The above-mentioned losses are integrated into an overall loss function, with several weights coefficients introduced: $w_{ds}$ for the deep survival loss, $w_{cl}$ for the contrastive loss, and $w_{int}$ for the three intermediate supervised losses. We aim to initially encourage the model to learn more about lesion, bag, or word category information for more efficient feature learning. As training progresses, the model should shift its focus towards patient-level objectives, such as $y_{i}$, $o_{i}$, and modal-agnostic features alignment. Hence, we defined $w_{int}$ as a dynamic weight, as follows:

$$w_{int}=0.1-0.1\times\exp\left( -5\times\left( 1-\frac{t}{T} \right)^{2} \right),$$

where $t$ and $T$ respectively denote the current epoch and the total number of epochs.

In summation, the overall loss function is expressed as:

$$l=l_{ce}+w_{ds}l_{ds}+w_{cl}l_{cl}+w_{int}\left( l_{r}+l_{pb}+l_{pw} \right).$$

For this study, both $w_{ds}$ and $w_{cl}$ were empirically set to 1.


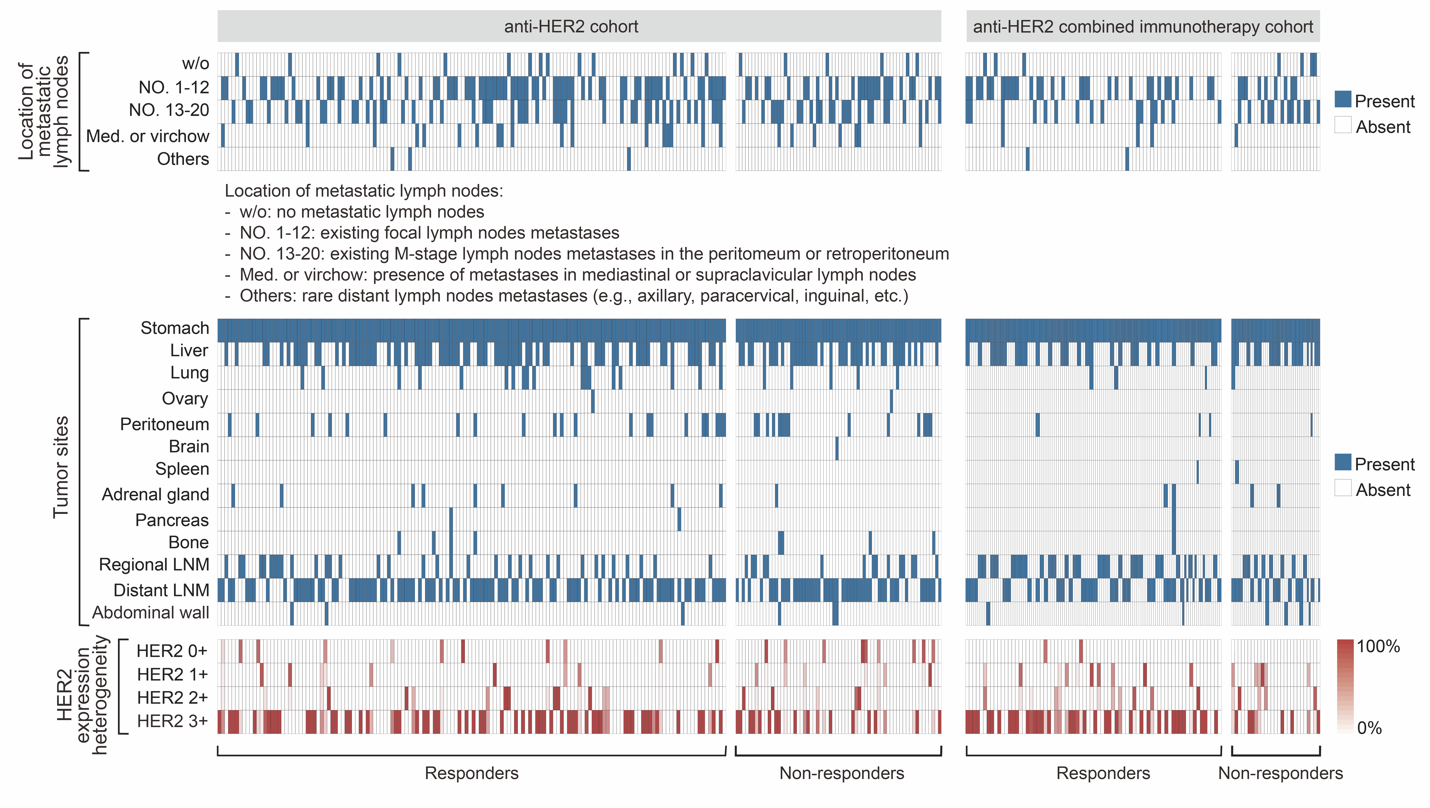


Figure. S1. Supplemental data characteristics.

Heatmap illustrating the distribution of clinical information with multiple labels, such as the location of metastatic lymph nodes, HER2 expression heterogeneity, and tumor sites, at the individual level. The heatmap was divided into three blocks, with each row indicating one category in the group, and each column representing one individual. For discrete data in blue blocks, such as the location of metastatic lymph nodes and tumor sites, filling blue represented presence, while white represented absence. For continuous data in red blocks, such as HER2 expression heterogeneity, the darker red represented a higher expression.


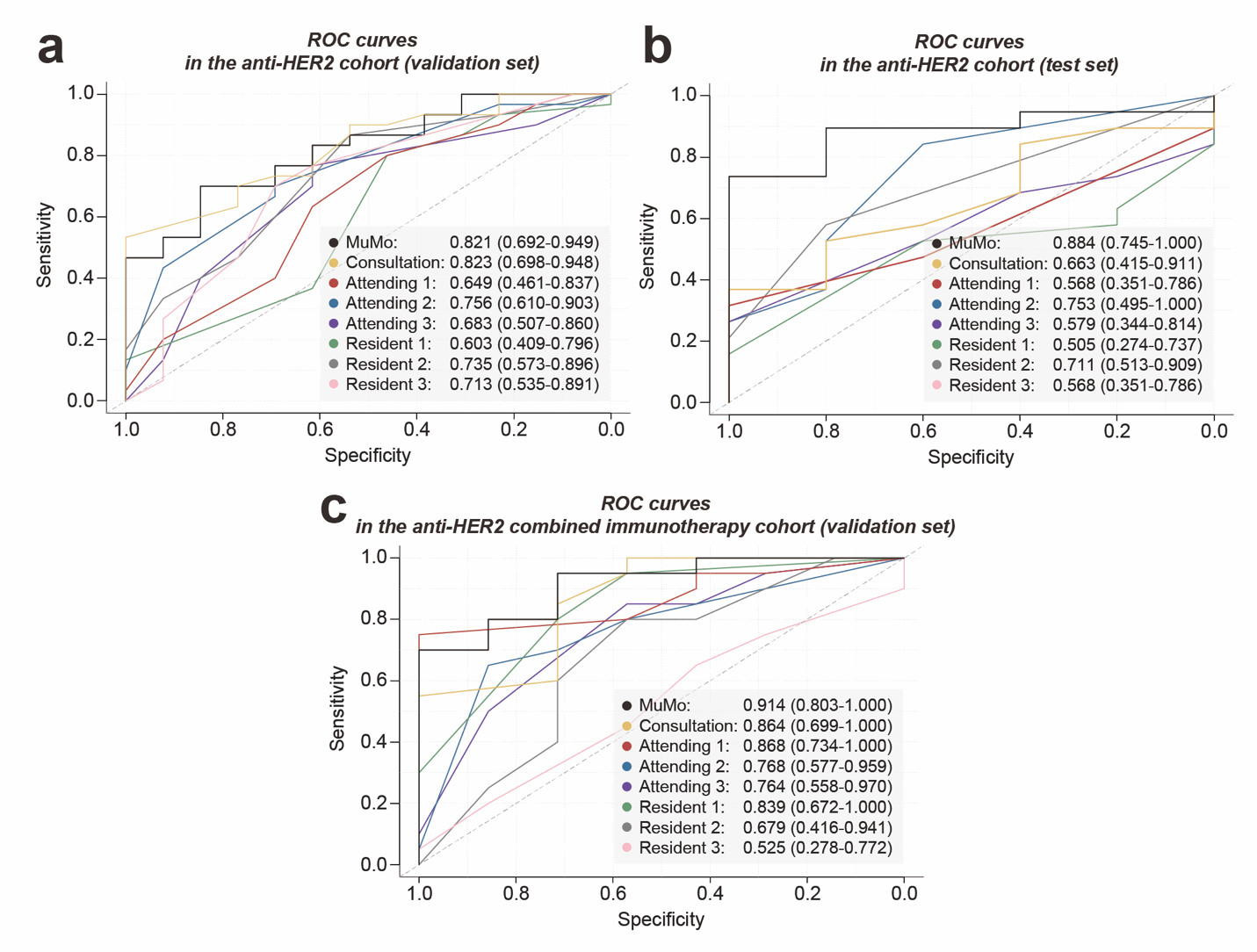


Figure. S2. Comparison of MuMo's predictive performance in treatment efficacy with that of six clinicians and their consultation.

**a–c** Comparison of MuMo with six clinicians on the anti-HER2 validation set, anti-HER2 test set, and anti-HER2 combined immunotherapy validation set, respectively.

We compared our proposed MuMo to six clinicians: three attending physicians and three residents. Each clinician was provided with the same baseline patient information for evaluating therapeutic response. The clinicians first predicted whether the patient would benefit from therapy based on their clinical experience and the provided data, followed by a quantitative benefit assessment on a 0-100% scale. To simulate a multi-clinician consultation, we averaged the scores of all six clinicians.


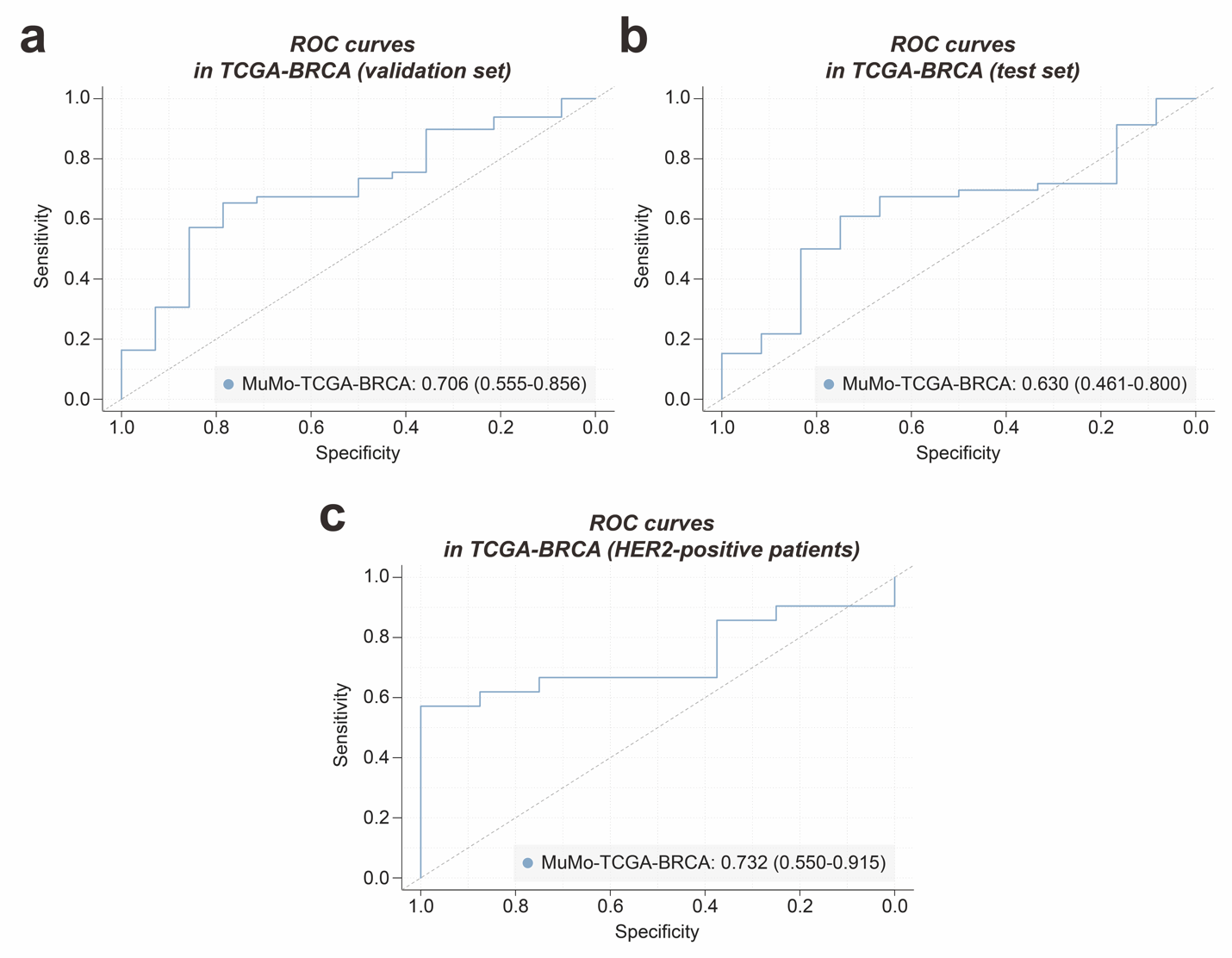


Figure. S3. Performance of the Multi-Modal Model (MuMo) in TCGA-BRCA dataset.

**a–c** Receiver operating characteristic (ROC) curves display MuMo’s performance in TCGA-BRCA validation set, test set, and HER2-positive patient set.


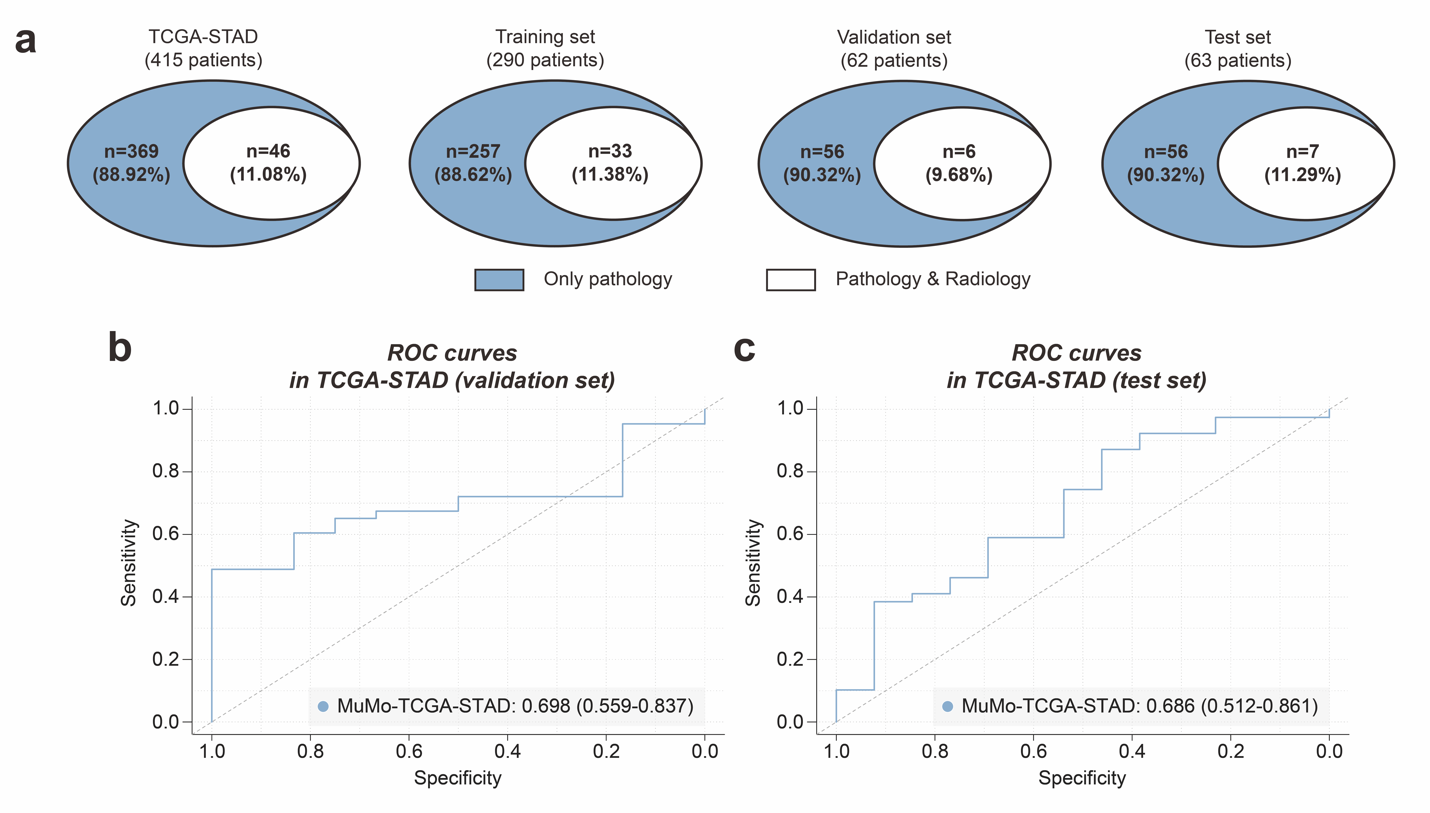


Figure. S4. Performance of the Multi-Modal Model (MuMo) in TCGA-STAD dataset.

**a** Distribution of proportions for pathological and radiological image data across different sets.

**b–c** Receiver operating characteristic (ROC) curves display MuMo’s performance in TCGA-BRCA validation set and test set.


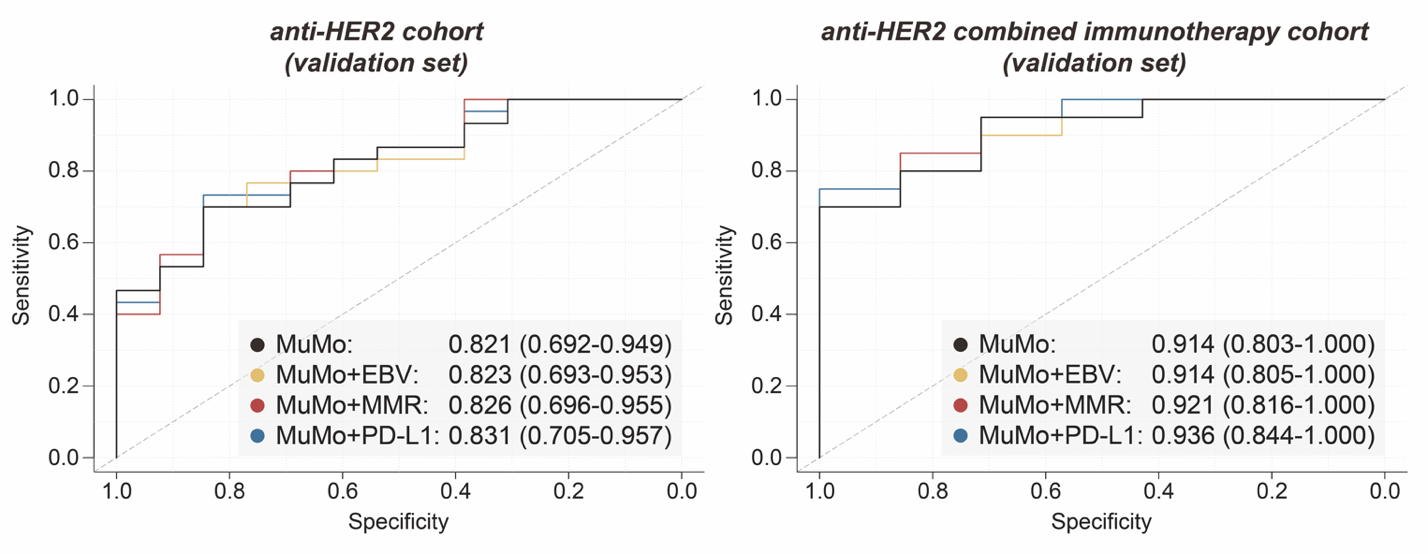


Figure. S5. ROC curves of MuMo for rapid expansion of molecular pathology data.

We encoded PD-L1, MMR, and EBV as new elements into the patient information sequence features without retraining MuMo. This validated MuMo's ability to rapidly incorporate new knowledge and highlighted the importance of molecular pathology data.


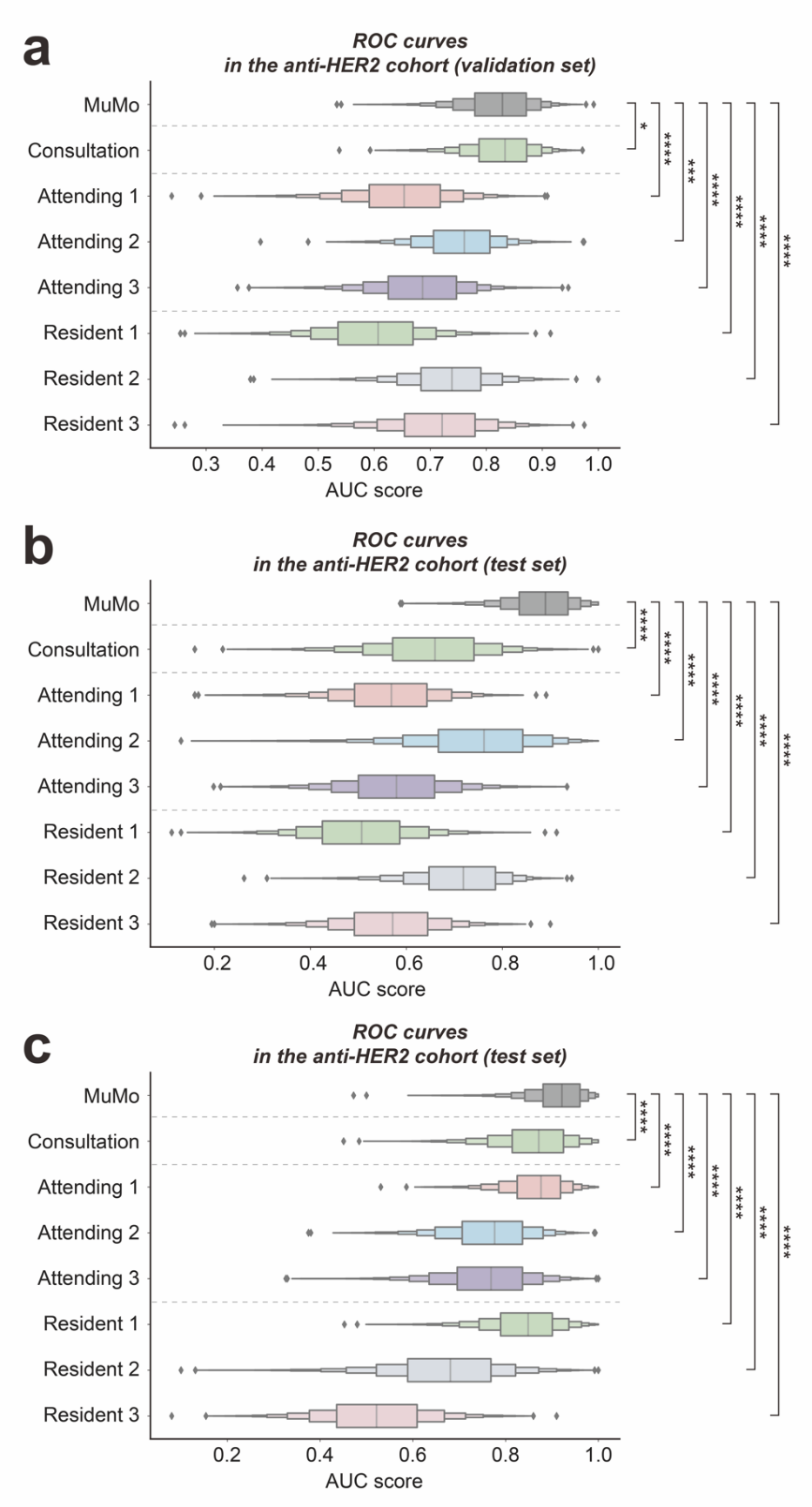


Figure. S6. Stability analysis of MuMo's predictive performance in treatment efficacy with that of four clinicians and their consultation.


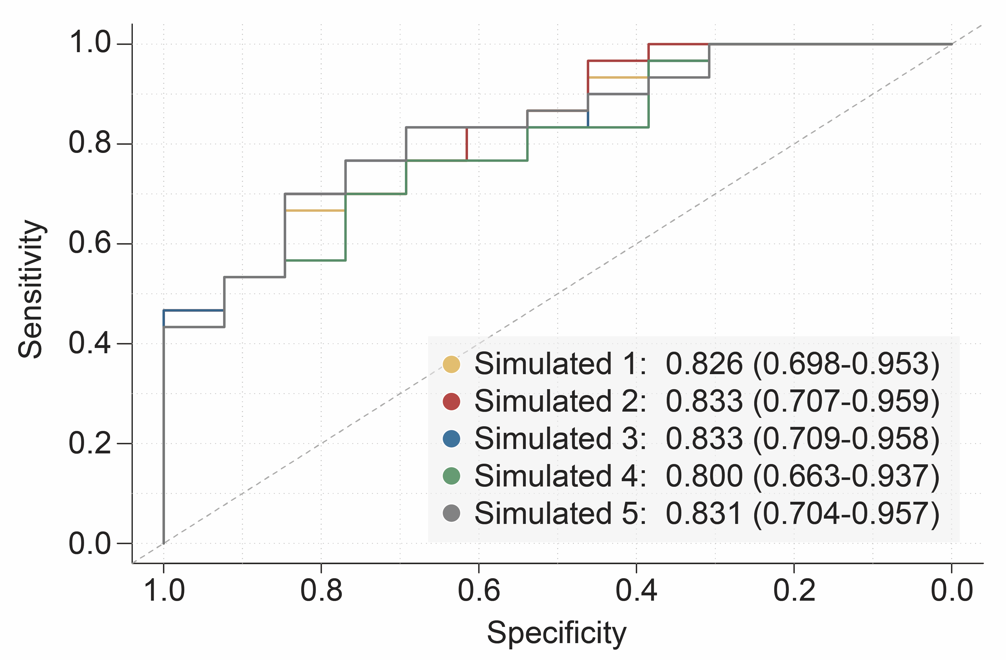


Figure. S7. **The impact of different clinician annotations on MuMo in the anti-HER2 validation set.**

Receiver operating characteristic (ROC) curves display MuMo’s performance with five different simulated inputs.


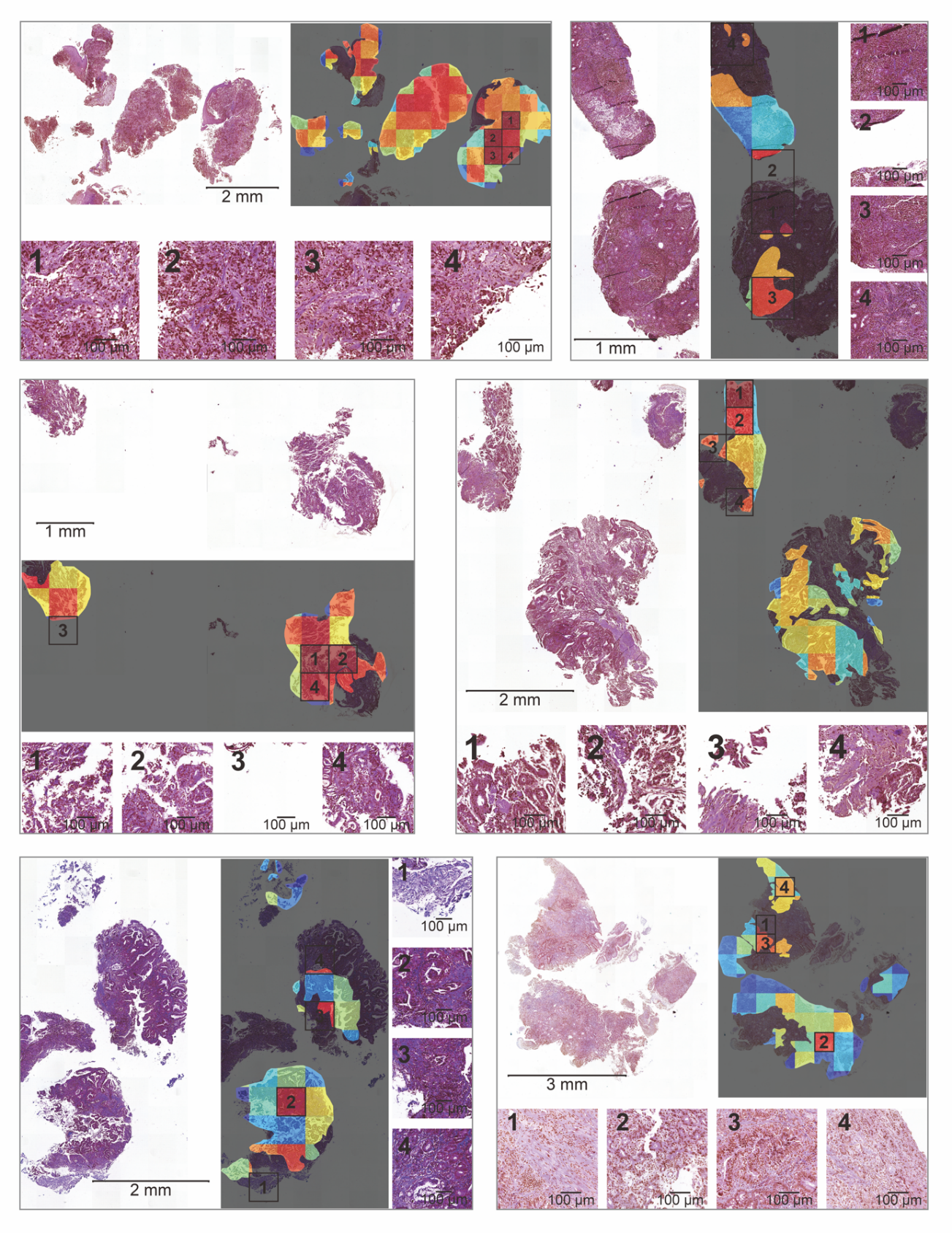


Figure. S8. **Additional visualization of important regions in pathology.**

Darker shades of red indicated a higher contribution to MuMo’s prediction, while darker shades of blue indicated a lower impact. The four patches below or to the right of each slice image represented the top four bags with the highest importance scores.

**
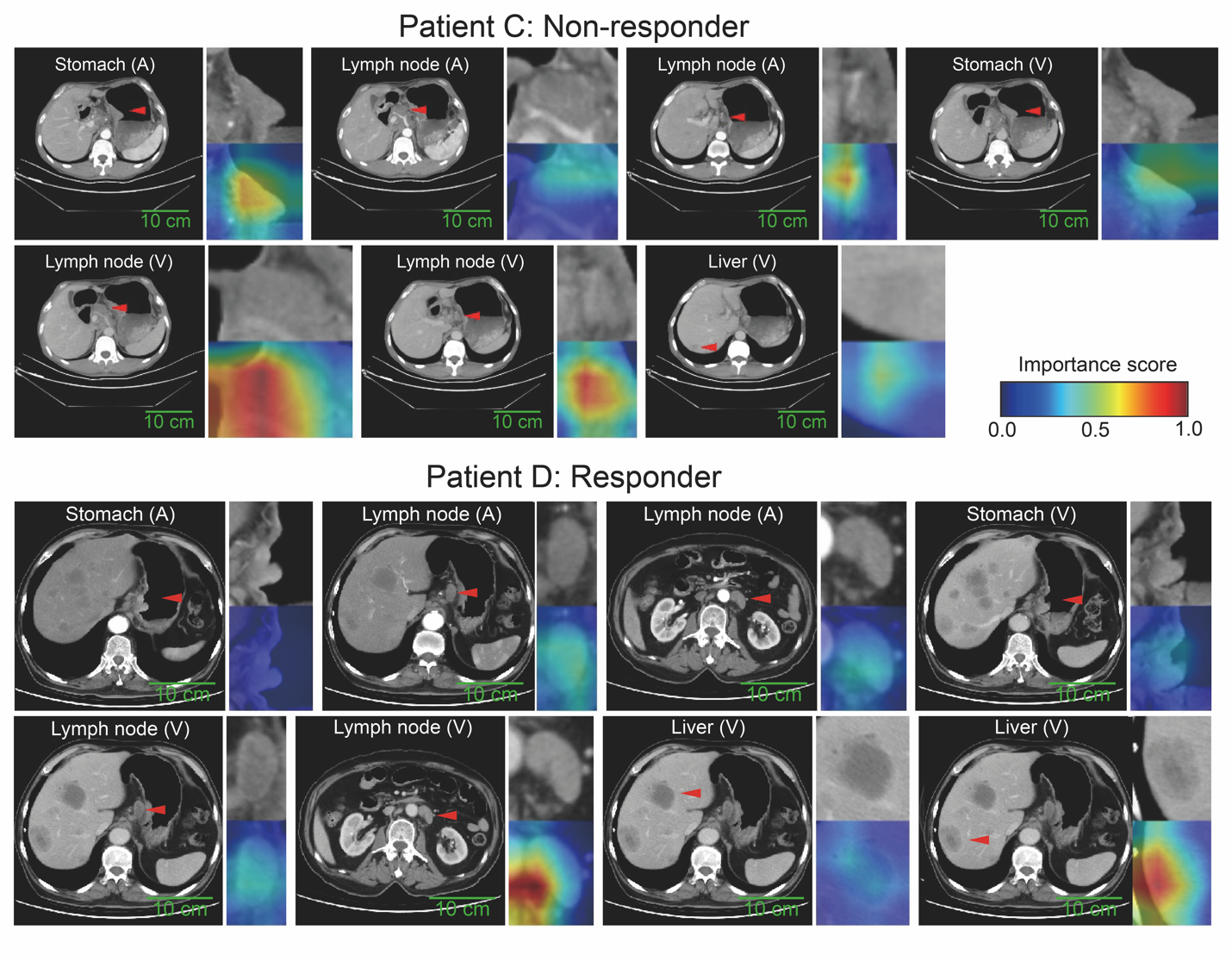
**

Figure. S9. **Additional visualization of important regions in radiology.**

The Grad-CAM algorithm highlighted regions of the input image that are important for the prediction made by the MuMo. Darker red regions indicated higher attention, while darker blue regions indicated lower attention.


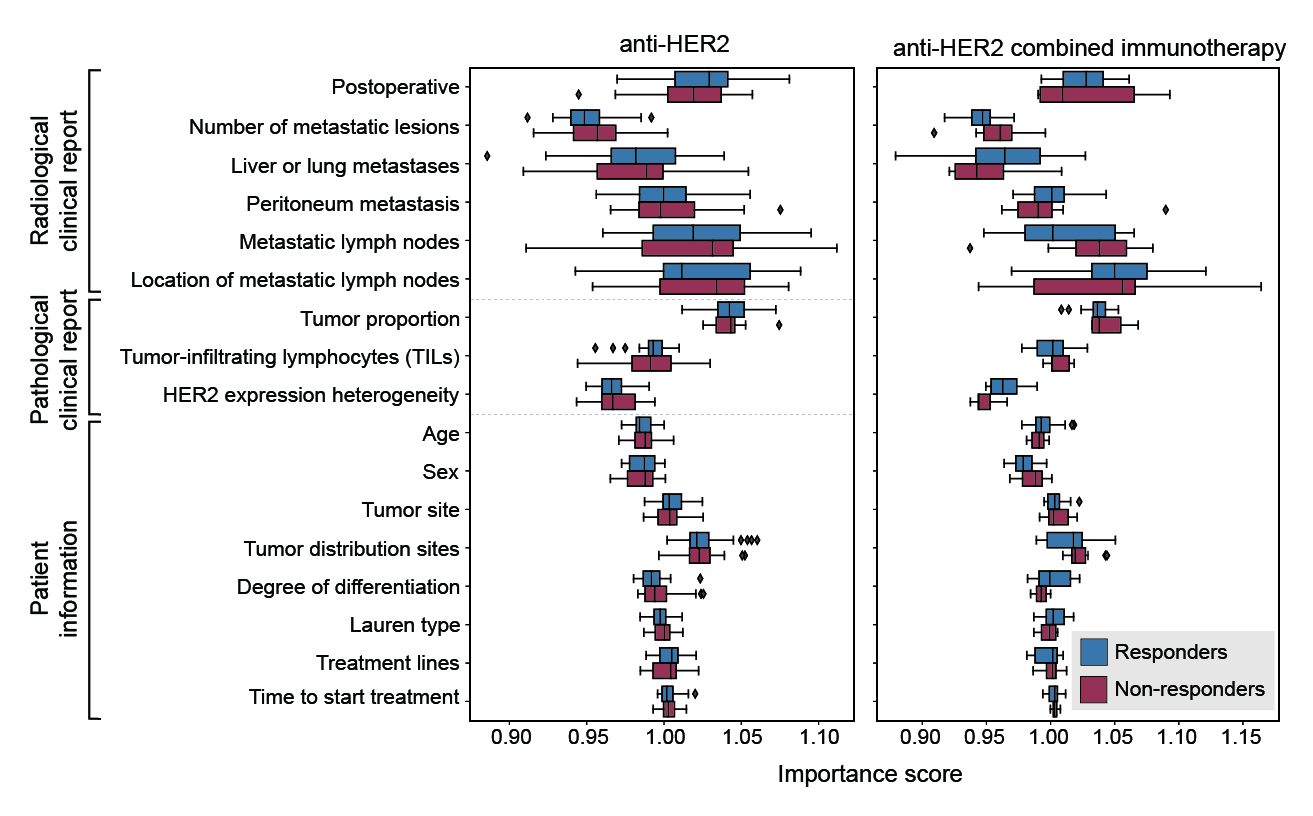


Figure. S10. **Visualization of the importance of different clinical information.**

The y-axis displayed the three groups of clinical information fused in the MuMo, including patient information (eight items), radiological structured clinical report (six items), and pathological structured clinical report (three items). The x-axis showed the importance scores of the different clinical information. Yellow and red colors indicated responders and non-responders, respectively.


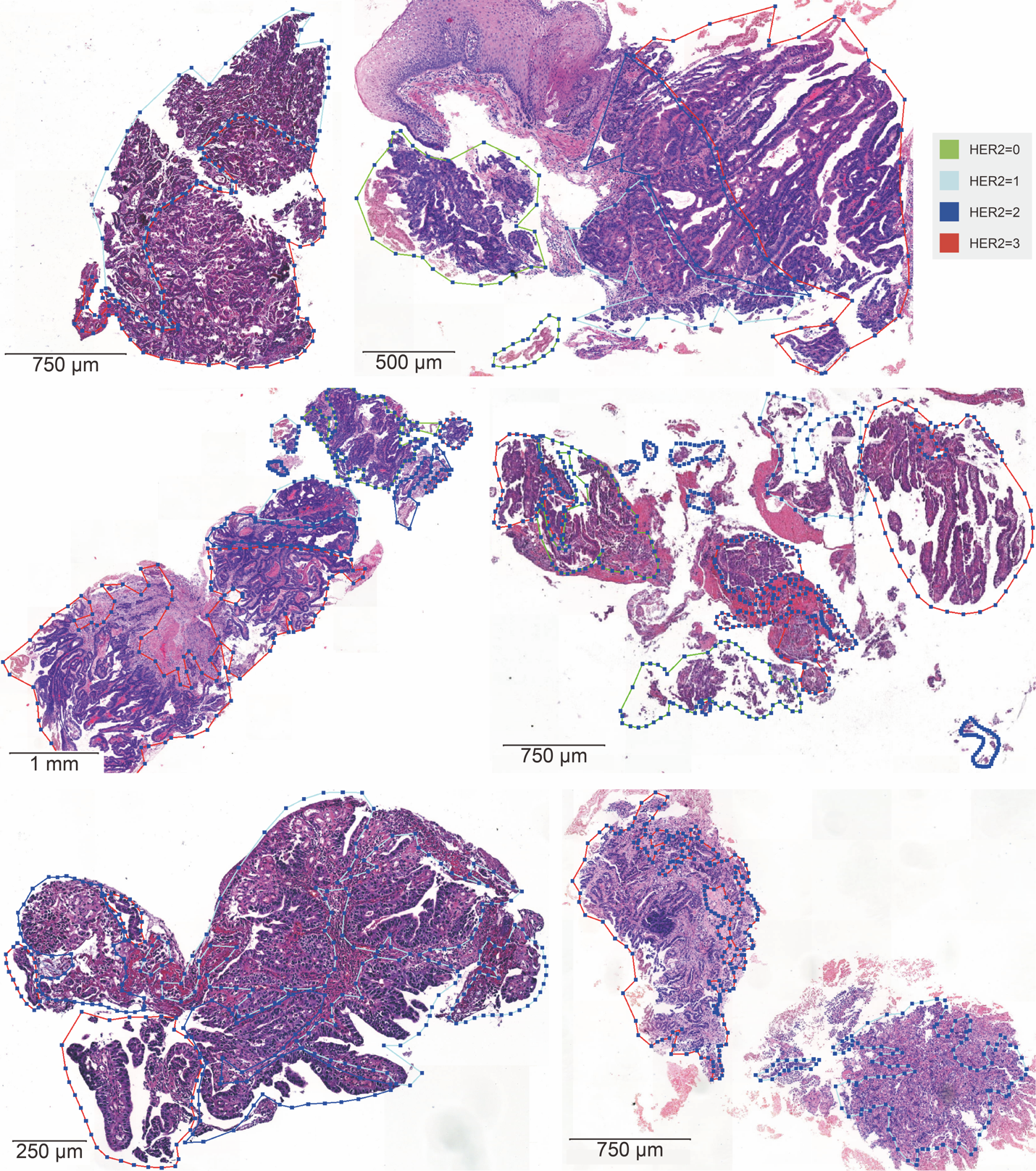


Figure. S11. **Visualization of annotated HER2 regions in pathological images.**


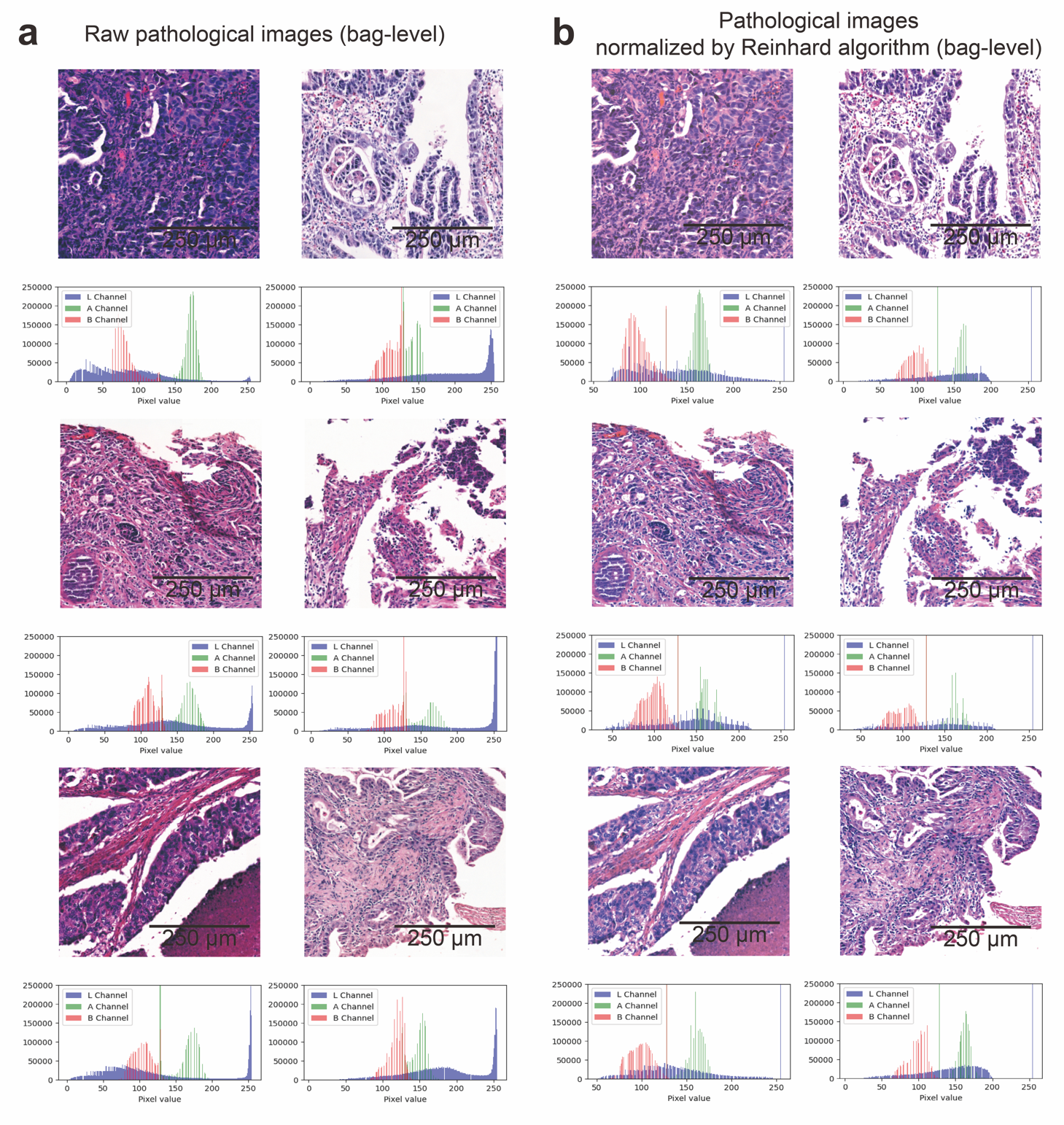


Figure. S12. **Comparative analysis of pathological images without and with color normalization based on Reinhard algorithm.**

**a** Raw bag-level pathological images. Each raw pathological image paired with a histogram representing the distribution of pixel values in the LAB color space. In the histograms, the L-component is depicted in blue, the A-component in green, and the B-component in red. The histograms illustrate pixel value distributions on the horizontal axis against their frequency on the vertical axis, with the y-axis scale fixed from 0 to 250,000.

**b** Corresponding normalized pathological images based on Reinhard algorithm.


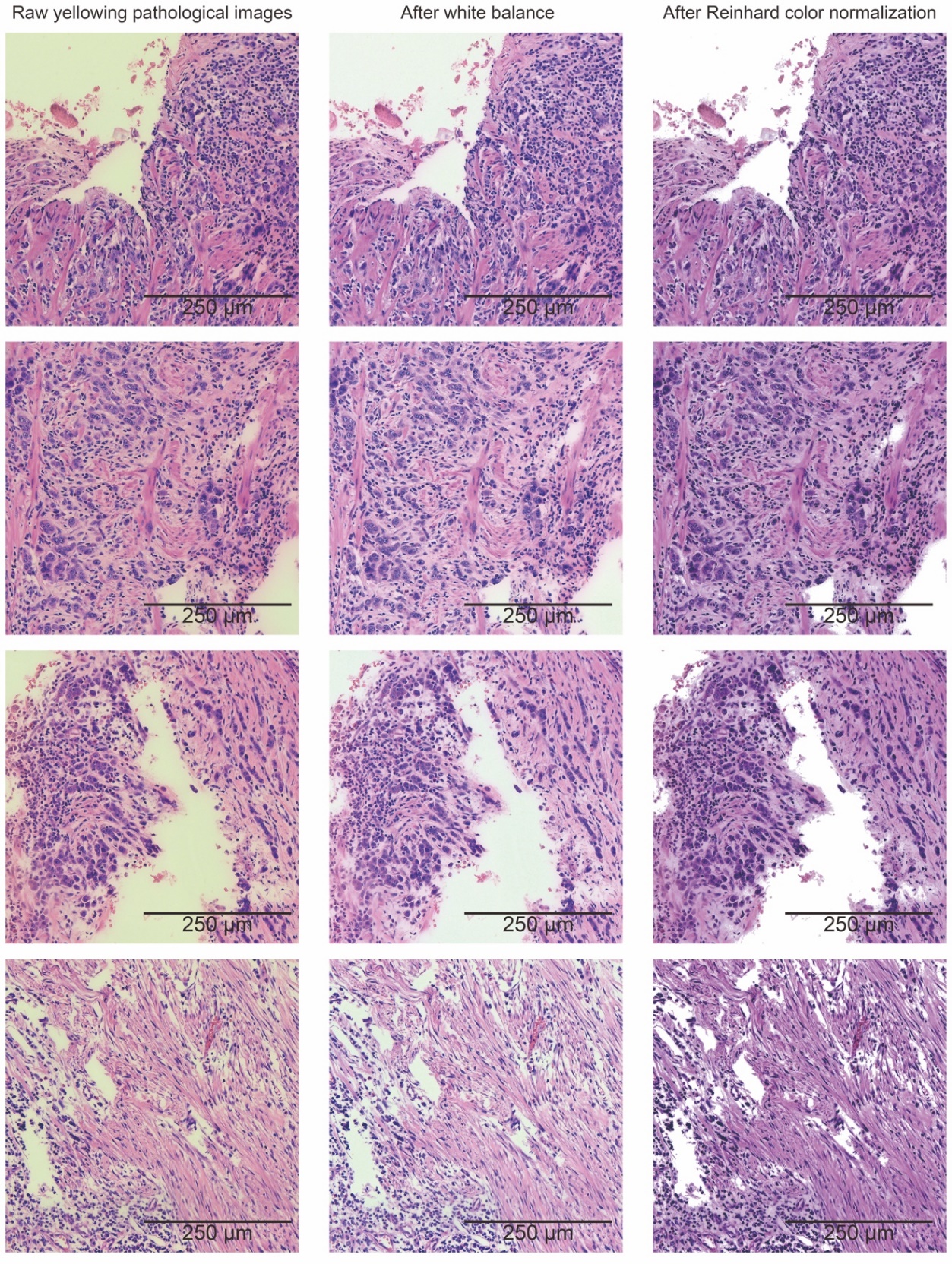


Figure. S13. **Comparative analysis of white balance processing on mildly yellowish pathological images.**

Each row represents a sample. The first column shows the original mildly yellowish image, the second column displays the image after white balance correction, and the third column presents the white-balanced image further with enhanced Reinhard color normalization.


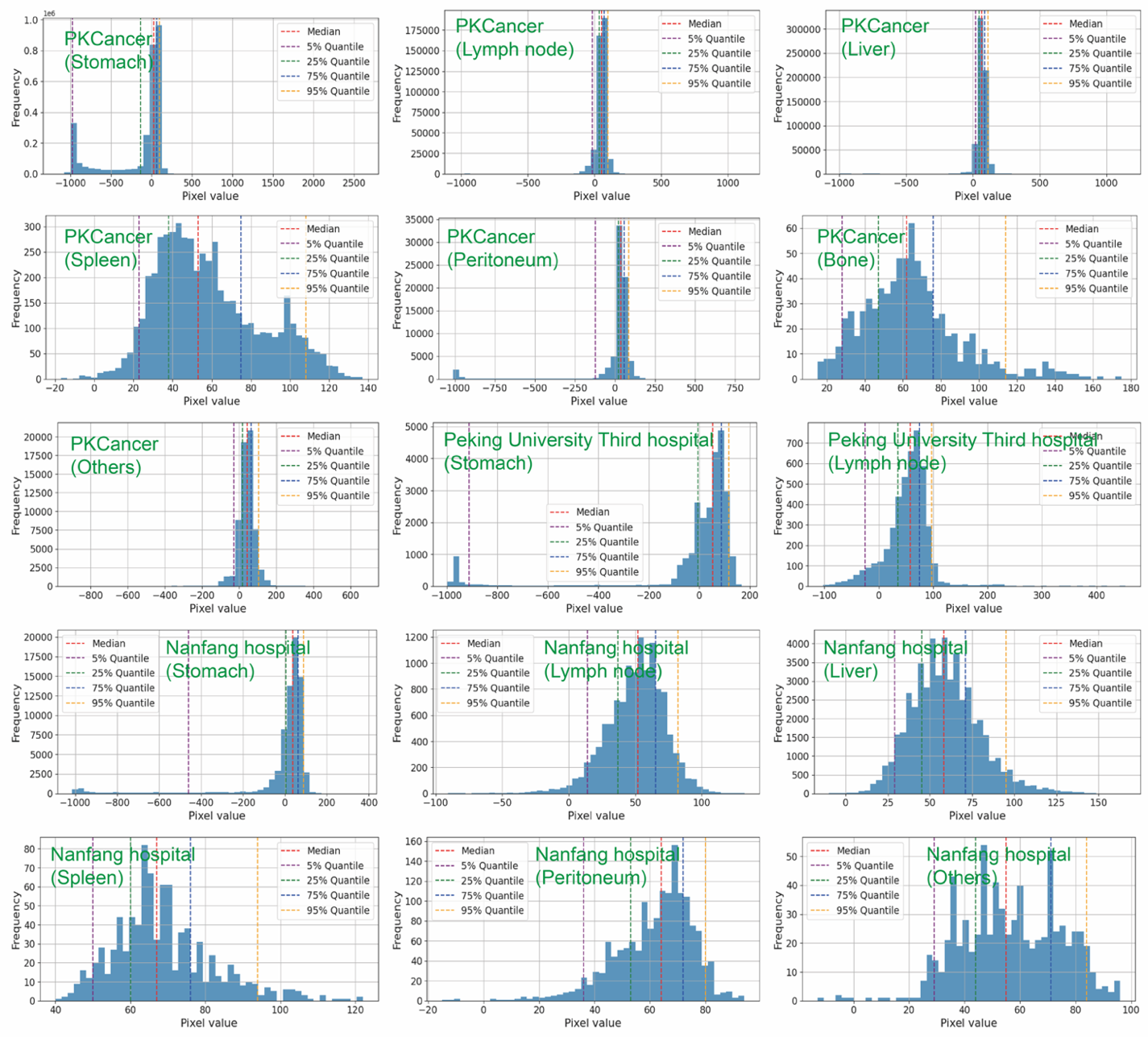


Figure. S14. **Multi-center distribution of CT HU values in various lesions.**

Each histogram corresponds to a specific center (indicated in green text) and represents the HU value distribution within a particular ROI delineated by radiologists (lesion type indicated in green text within parentheses). The x-axis denotes the HU values, while the y-axis indicates the frequency of occurrence. The dashed lines represent percentiles: red for the median, purple for the 5th, green for the 25th, blue for the 75th, and yellow for the 95th.


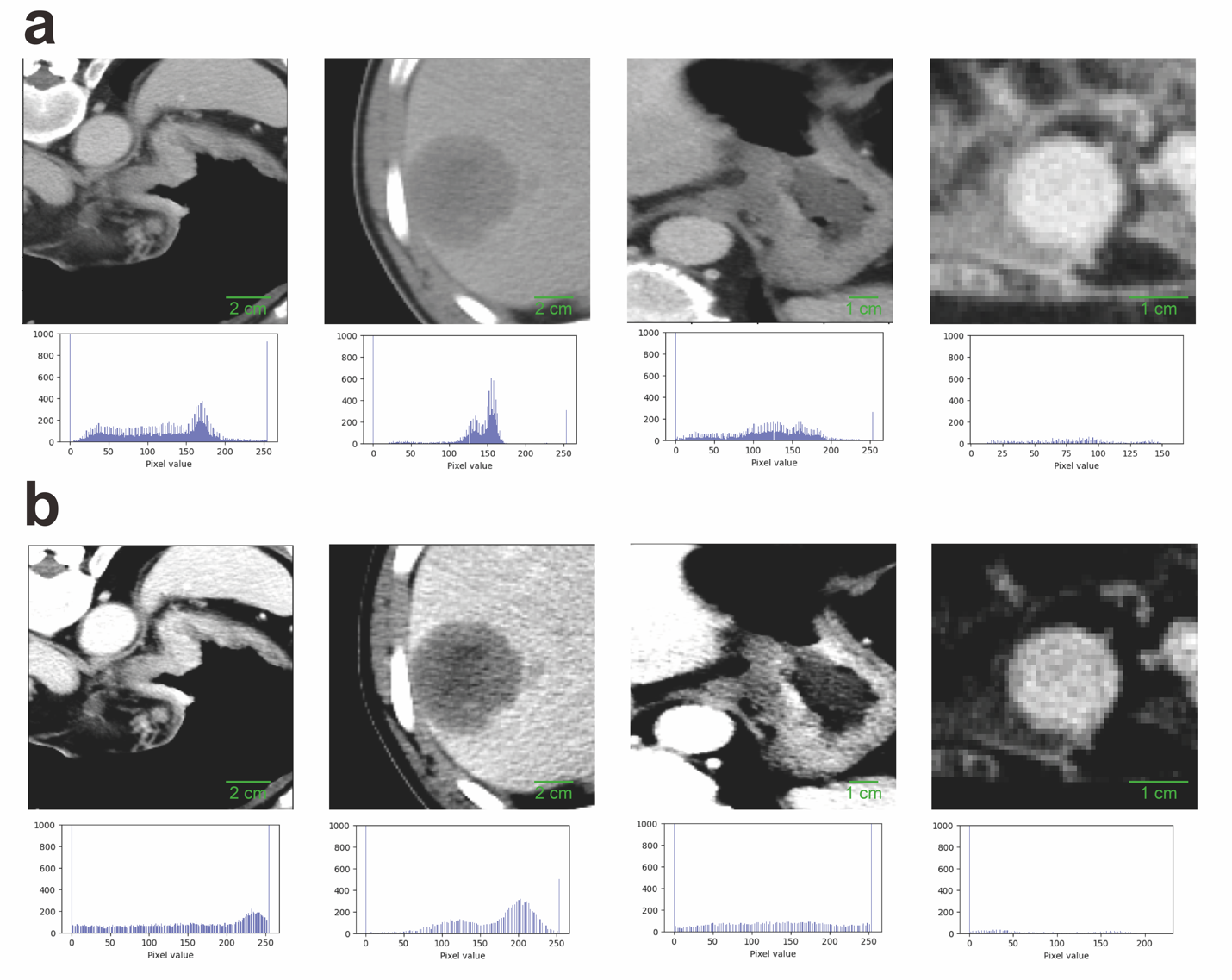


Figure. S15. **Comparative analysis of radiological ROI images without and with normalization via dynamic window levels and widths.**

**a** Standardization using a fixed window-level of 50 and window-width of 350 for all lesions from any center. Each ROI image is accompanied by its pixel value distribution, with the x-axis representing pixel values (gray-scale) and the y-axis representing frequency.

**b** Dynamic normalization using pre-defined window-levels and window-widths from Supplementary Figure S14 during radiologist review and annotation stage, differentiating between centers and lesions (as detailed in Supplementary Table S10).


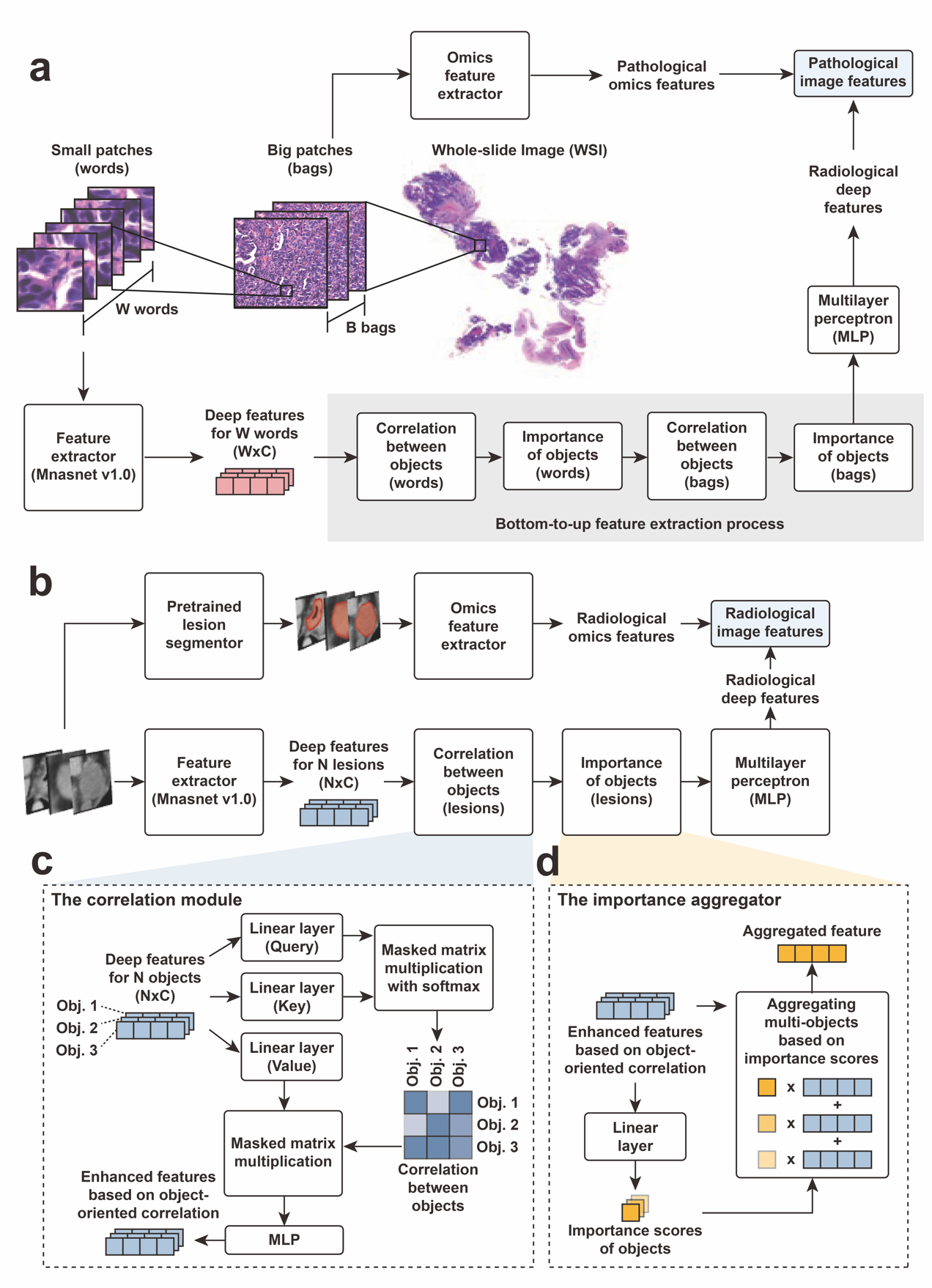


Figure. S16. **Details of the feature extraction process within MuMo.**

**a** Extracting pathological image features, consisting of pathological deep features and pathological omics features. **b** Extracting radiological image features, consisting of deep and omics features. **c** Details of the correlation module. **d** Details of the importance aggregator.


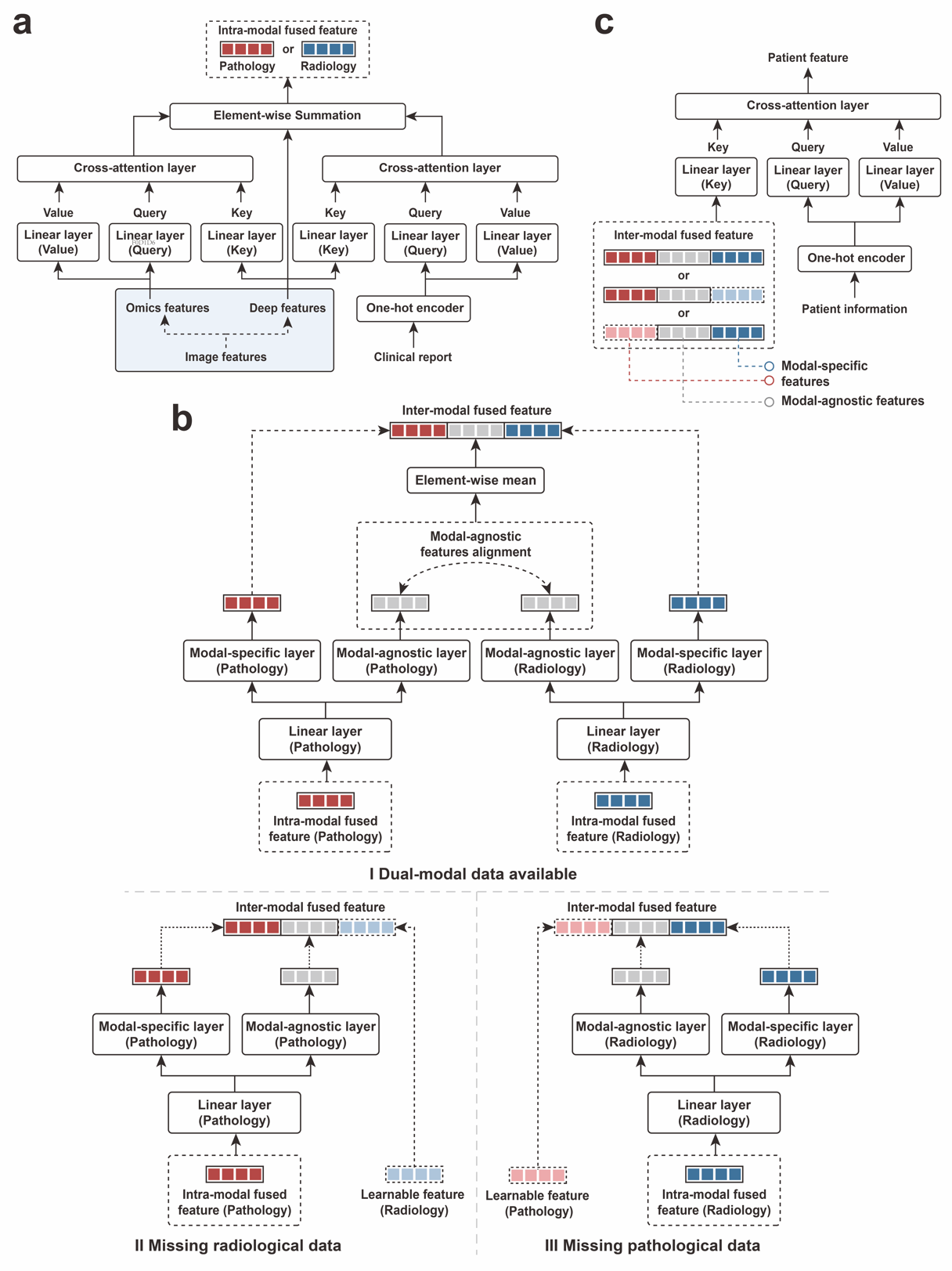


Figure. S17. **Details of three fusion modules within MuMo.**

**a** Intra-modal fusion module was used for fusing information within radiology or pathology, such as image features and clinical reports. **b** Inter-modal fusion module was responsible for fusing radiological and pathological features. Different fusion strategies were designed to adapt to three different situations in clinical practice. **c** Patient information fusion module enhanced the features with patient information, to produce the final patient-level features.


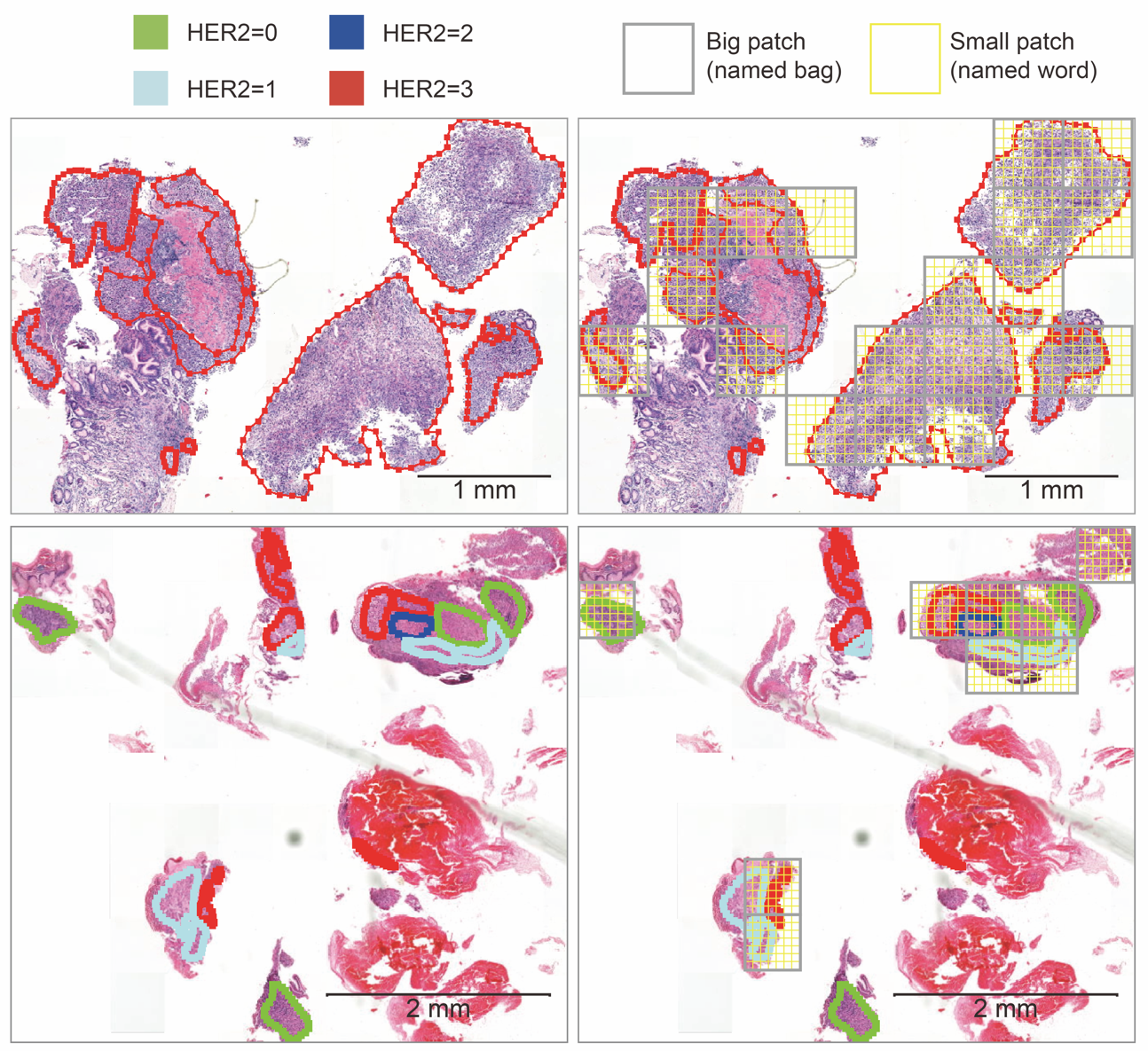


Figure. S18. **Visualization of pathological images preprocessing workflow.**

The left side of the figure displays the whole-slide image along with the annotations provided by the pathologist. These annotations are color-coded in accordance with their HER2 expression statuses, including HER2=0, HER2=1+, HER2=2+, and HER2=3+. The right side of the figure shows the pre-processing workflow utilized in this study. Initially, large patches, referred to as “bags”, are automatically selected if the annotated region comprises more than 25% of the area. Subsequently, these “bags” undergo further partitioned into smaller patches, referred to as “words”. These “words” then function as the input for the proposed MuMo for feature extraction.


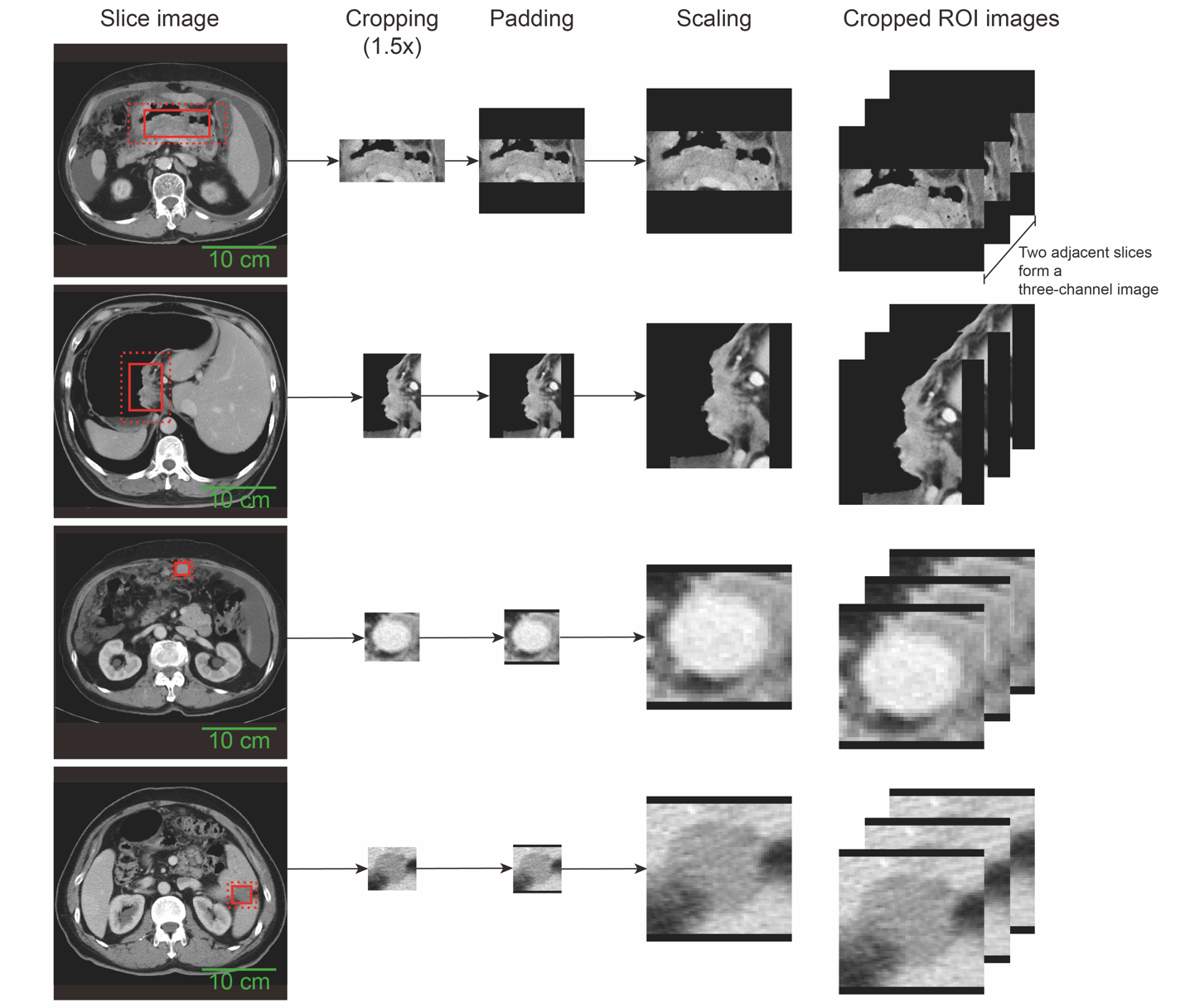


Figure. S19**. Visualization of radiological images preprocessing workflow.**

The bounding boxes provided by the radiologist, delineated by the solid red rectangle, are scaled up by a factor of 1.5 (dashed red rectangle) as a basis for cropping, ensuring the incorporation of relevant contextual information. Subsequently, these cropped images undergo zero padding to create a minimum bounding square. Following this step, the images are resized to a resolution of 112$\times$112 pixels. Each image is then paired with its immediate superior and inferior slices, yielding a three-channel image, called the cropped region of interest (ROI) image in our study, which is then used as input to our model.

Figure. S20. **Illustration of the pre-trained lesion segmenter’s results.**

The red line represents the edge of the segmentation result, and the area surrounded by it is the segmentation result. The label “#1” signifies the use of the stomach lesion segmenter, whereas “#2” denotes the use of the metastatic lesion segmenter.

Table S1. Baseline characteristics of training and validation sets in anti-HER2 cohort.

| Characteristic | Training set (n=215) | Validation set (n=56) |
| --- | --- | --- |
| Age |  |  |
| Median, IQR | 63, 55-69 | 63, 56-68 |
| Sex |  |  |
| Male | 177 (82.33%) | 48 (85.71%) |
| Female | 38 (17.67%) | 8 (14.29%) |
| Tumor site |  |  |
| GEJ | 67 (31.16%) | 15 (26.79%) |
| Non-GEJ | 148 (68.84%) | 41 (73.21%) |
| Differentiation |  |  |
| Low | 100 (46.51 %) | 29 (51.79%) |
| Mid | 107 (49.77%) | 25 (44.64%) |
| High | 8 (3.72%) | 2 (3.57%) |
| Lauren type |  |  |
| Intestinal | 155 (72.09 %) | 39 (69.64%) |
| Diffused | 25 (11.63%) | 9 (16.07%) |
| Mixed | 35 (16.28%) | 8 (14.29%) |
| PD-L1 expression |  |  |
| Positive | 33 (15.35%) | 11 (19.64%) |
| Negative | 48 (22.33%) | 12 (21.43%) |
| N/A | 134 (62.33%) | 33 (58.93%) |
| MMR status |  |  |
| pMMR | 107 (49.77%) | 30 (53.57%) |
| dMMR | 2 (0.93%) | 0 (0.00%) |
| N/A | 106 (49.30%) | 26 (46.43%) |
| EBV status |  |  |
| Positive | 2 (0.93%) | 1 (1.79%) |
| Negative | 94 (43.72%) | 25 (44.64%) |
| N/A | 119 (55.35%) | 30 (53.57%) |

Table S2. Baseline characteristics of training and validation sets in anti-HER2 with combined immunotherapy cohort.

| Characteristic | Training set (n=89) | Validation set (n=30) |
| --- | --- | --- |
| Age |  |  |
| Median, IQR | 65, 59-70 | 65, 56-73 |
| Sex |  |  |
| Male | 72 (80.90%) | 23 (76.67%) |
| Female | 17 (19.10%) | 7 (23.33%) |
| Tumor site |  |  |
| GEJ | 26 (29.21%) | 9 (30.00%) |
| Non-GEJ | 63 (70.79%) | 21 (70.00%) |
| Differentiation |  |  |
| Low | 43 (48.31%) | 18 (60.00 %) |
| Mid | 45 (50.56 %) | 12 (40.00%) |
| High | 1 (1.12%) | 0 (0.00%) |
| Lauren type |  |  |
| Intestinal | 69 (77.53%) | 22 (73.33%) |
| Diffused | 7 (7.87%) | 5 (16.67%) |
| Mixed | 13 (14.61%) | 3 (10.00%) |
| PD-L1 expression |  |  |
| Positive | 29 (32.58%) | 14 (46.67%) |
| Negative | 19 (21.35%) | 6 (20.00%) |
| N/A | 41 (46.07%) | 10 (33.33%) |
| MMR status |  |  |
| pMMR | 77 (86.52%) | 27 (90.00%) |
| dMMR | 0 (0.00%) | 1 (3.33%) |
| N/A | 12 (13.48%) | 2 (6.67%) |
| EBV status |  |  |
| Positive | 1 (1.12%) | 0 (0.00%) |
| Negative | 64 (71.91%) | 25 (83.33%) |
| N/A | 24 (26.97%) | 5 (16.67%) |

Table S3. The summary performance of clinicians and MuMo.

These results were evaluated on the validation sets in anti-HER2 and anti-HER2 combined immunotherapy cohorts.

|  | AUC | 95%CI lower | 95%CI upper |
| --- | --- | --- | --- |
| Anti-HER2 cohort (validation set) | | | |
| Attending 1 | 0.649 | 0.461 | 0.837 |
| Attending 2 | 0.756 | 0.610 | 0.903 |
| Attending 3 | 0.683 | 0.507 | 0.860 |
| Resident 1 | 0.603 | 0.409 | 0.796 |
| Resident 2 | 0.735 | 0.573 | 0.896 |
| Resident 3 | 0.713 | 0.535 | 0.891 |
| Consultation | **0.823** | 0.698 | 0.948 |
| MuMo | 0.821 | 0.692 | 0.949 |
| Anti-HER2 cohort (test set) | | | |
| Attending 1 | 0.568 | 0.351 | 0.786 |
| Attending 2 | 0.753 | 0.495 | 1.000 |
| Attending 3 | 0.579 | 0.344 | 0.814 |
| Resident 1 | 0.505 | 0.274 | 0.737 |
| Resident 2 | 0.711 | 0.513 | 0.909 |
| Resident 3 | 0.568 | 0.351 | 0.786 |
| Consultation | 0.663 | 0.415 | 0.911 |
| MuMo | **0.884** | 0.745 | 1.000 |
| Anti-HER2 combined immunotherapy cohort (validation set) | | | |
| Attending 1 | 0.868 | 0.734 | 1.000 |
| Attending 2 | 0.768 | 0.577 | 0.959 |
| Attending 3 | 0.764 | 0.558 | 0.970 |
| Resident 1 | 0.839 | 0.672 | 1.000 |
| Resident 2 | 0.679 | 0.416 | 0.941 |
| Resident 3 | 0.525 | 0.278 | 0.772 |
| Consultation | 0.864 | 0.699 | 1.000 |
| MuMo | **0.914** | 0.803 | 1.000 |

**Bold** indicates the best performance.

Underline indicates suboptimal performance.

Table S4. Performance of Number Needed to Treat (NNT).

These results were evaluated on the validation sets in anti-HER2 cohorts.

|  | NNT | 95%CI lower | 95%CI upper |
| --- | --- | --- | --- |
| Attending 1 | 4.02 | -5.90 | 134.21 |
| Attending 2 | 2.55 | 1.51 | 19.55 |
| Attending 3 | 2.62 | 1.43 | 12.59 |
| Resident 1 | 3.82 | -13.92 | 55.00 |
| Resident 2 | 2.47 | 1.35 | 8.61 |
| Resident 3 | 2.55 | 1.45 | 11.74 |
| Consultation | 2.13 | 1.39 | 7.59 |
| MuMo | **1.83** | 1.29 | 4.24 |

**Bold** indicates the best performance.

Underline indicates suboptimal performance.

Table S5. **Consistency measurement between five simulated doctor sketching and original doctor sketching.**

| Region | Simulated  sketching 1 | Simulated  sketching 2 | Simulated  sketching 3 | Simulated  sketching 4 | Simulated  sketching 5 |
| --- | --- | --- | --- | --- | --- |
| Pathology |  |  |  |  |  |
| HER2=0 | 0.748 | 0.732 | 0.739 | 0.759 | 0.733 |
| HER2=1+ | 0.843 | 0.850 | 0.848 | 0.855 | 0.844 |
| HER2=2+ | 0.824 | 0.824 | 0.820 | 0.831 | 0.824 |
| HER2=3+ | 0.823 | 0.824 | 0.828 | 0.820 | 0.823 |
| Average | 0.809 | 0.808 | 0.809 | 0.816 | 0.806 |
| Radiology |  |  |  |  |  |
| Stomach | 0.838 | 0.863 | 0.849 | 0.847 | 0.836 |
| Liver | 0.701 | 0.692 | 0.672 | 0.680 | 0.669 |
| Lymph node | 0.684 | 0.663 | 0.652 | 0.677 | 0.688 |
| Peritoneum | 0.672 | 0.728 | 0.629 | 0.678 | 0.663 |
| Others | 0.656 | 0.711 | 0.681 | 0.662 | 0.627 |
| Average | 0.710 | 0.731 | 0.697 | 0.709 | 0.696 |

* The consistency measurement is based on the Dice coefficient, which is a statistic used to measure the similarity between sets and defined as $D=2\left| X\cap Y \right|/(\left| X \right|+\left| Y \right|)$. The coefficient ranges from 0 (no similarity) to 1 (perfect similarity).

Table S6. **The experiment of the integration of pathology and radiology.**

These results were evaluated on a subset of patients who possessed both radiological and pathological data in the anti-HER2 validation set.

|  | AUC | 95%CI lower | 95%CI upper |
| --- | --- | --- | --- |
| The radiology-only model | 0.639 | 0.369 | 0.908 |
| The pathology-only model | 0.703 | 0.334 | 1.000 |
| MuMo | **0.750** | 0.499 | 1.000 |

**Bold** indicates the best performance.

Table S7. **The experiment of the integration modes.**

These results were evaluated on the validation sets in anti-HER2 cohorts.

|  | AUC | 95%CI lower | 95%CI upper |
| --- | --- | --- | --- |
| Element-wise Multiplication | 0.577 | 0.377 | 0.777 |
| Summation | 0.682 | 0.512 | 0.852 |
| Concatenation | 0.731 | 0.579 | 0.883 |
| MuMo w/o alignment | 0.772 | 0.632 | 0.912 |
| MuMo | **0.821** | 0.692 | 0.949 |

**Bold** indicates the best performance.

"w/o": This is an abbreviation for the term "without".

Table S8. **The experiment of the inclusion of clinical information.**

These results were evaluated on the validation sets in anti-HER2 cohorts.

|  | AUC | 95%CI lower | 95%CI upper |
| --- | --- | --- | --- |
| without any clinical information | 0.703 | 0.539 | 0.866 |
| only with clinical reports | 0.769 | 0.624 | 0.914 |
| MuMo | **0.821** | 0.692 | 0.949 |

**Bold** indicates the best performance.

Table S9**.** **Area proportions of varying HER2 expression levels across three cohorts.**

| Region | All | Anti-HER2  cohort | Anti-HER2  combined immunotherapy  cohort | External  cohort |
| --- | --- | --- | --- | --- |
| HER2=0 | \| 6.67% \| \| --- \| | 8.75% | 1.58% | 15.94% |
| HER2=1+ | 14.06% | 14.96% | 13.94% | 7.62% |
| HER2=2+ | 19.18% | 6.90% | 33.94% | 41.14% |
| HER2=3+ | 60.09% | 69.39% | 50.55% | 35.29% |

Table S10**.** **Area proportions of varying HER2 expression levels across three cohorts.**

The dynamic window-levels and window-widths were confirmed during the review and annotation stage by radiologists based on Supplementary Figure S14. These settings were used to normalize the radiological ROI images to mitigate the visual domain shift caused by different HU value distributions across centers and lesions (Supplementary Figure S15).

| Medical center | Window level | Window width |
| --- | --- | --- |
| Peking Cancer Hospital (PKCancer) |  |  |
| Stomach | 30 | 100 |
| Liver | 60 | 140 |
| Lymph node | 50 | 120 |
| Spleen | 50 | 100 |
| Peritoneum | 40 | 100 |
| Bone | 60 | 70 |
| Others | 40 | 120 |
| Nanfang Hospital |  |  |
| Stomach | 40 | 100 |
| Liver | 60 | 100 |
| Lymph node | 50 | 80 |
| Spleen | 65 | 70 |
| Peritoneum | 65 | 50 |
| Others | 55 | 60 |
| Peking University Third Hospital |  |  |
| Stomach | 50 | 130 |
| Lymph node | 60 | 80 |

Table S11**.** **The pathological parameterless encoder.**

This pre-defined parameterless encoder was used to map pathological structured clinical reports into embeddings.

| Characteristic | Embedding |
| --- | --- |
| Tumor proportion |  |
| 0% | [0, 0, 0, 0, 0, 0, 0, 0, 0, 0] |
| 10% | [1, 0, 0, 0, 0, 0, 0, 0, 0, 0] |
| 20% | [0, 1, 0, 0, 0, 0, 0, 0, 0, 0] |
| 30% | [0, 0, 1, 0, 0, 0, 0, 0, 0, 0] |
| 40% | [0, 0, 0, 1, 0, 0, 0, 0, 0, 0] |
| 50% | [0, 0, 0, 0, 1, 0, 0, 0, 0, 0] |
| 60% | [0, 0, 0, 0, 0, 1, 0, 0, 0, 0] |
| 70% | [0, 0, 0, 0, 0, 0, 1, 0, 0, 0] |
| 80% | [0, 0, 0, 0, 0, 0, 0, 1, 0, 0] |
| 90% | [0, 0, 0, 0, 0, 0, 0, 0, 1, 0] |
| 100% | [0, 0, 0, 0, 0, 0, 0, 0, 0, 1] |
| Tumor-infiltrating lymphocytes (TILs) |  |
| 0.0 | [0, 0, 0, 0, 0, 0, 0, 0, 0, 0] |
| 0.1 | [1, 0, 0, 0, 0, 0, 0, 0, 0, 0] |
| 0.2 | [0, 1, 0, 0, 0, 0, 0, 0, 0, 0] |
| 0.3 | [0, 0, 1, 0, 0, 0, 0, 0, 0, 0] |
| 0.4 | [0, 0, 0, 1, 0, 0, 0, 0, 0, 0] |
| 0.5 | [0, 0, 0, 0, 1, 0, 0, 0, 0, 0] |
| 0.6 | [0, 0, 0, 0, 0, 1, 0, 0, 0, 0] |
| 0.7 | [0, 0, 0, 0, 0, 0, 1, 0, 0, 0] |
| 0.8 | [0, 0, 0, 0, 0, 0, 0, 1, 0, 0] |
| 0.9 | [0, 0, 0, 0, 0, 0, 0, 0, 1, 0] |
| 1.0 | [0, 0, 0, 0, 0, 0, 0, 0, 0, 1] |
| HER2 expression heterogeneity* |  |
| HER2 0-3 percentage proportion | [HER2-0%, HER2-1%, HER2-2%, HER2-3%] |

* The heterogeneity of HER2 expression, such as (0+40%, 1+30%, 2+30%, 3+0%), is represented by an embedding of length 4. Each value in this embedding corresponds to the percentage, accurate to two decimal places, of its associated expression level. For instance, (0+40%, 1+30%, 2+30%, 3+0%) is converted to [0.40, 0.30, 0.30, 0.00].

Table S12**. The radiological parameterless encoder.**

This parameterless encoder was used to map radiological structured clinical reports into embeddings.

| Characteristic | Embedding |
| --- | --- |
| Postoperative |  |
| No | [1, 0] |
| Yes | [0, 1] |
| Number of metastatic lesions |  |
| 0 | [1, 0, 0, 0, 0, 0, 0] |
| 1 | [0, 1, 0, 0, 0, 0, 0] |
| 2 | [0, 0, 1, 0, 0, 0, 0] |
| 3 | [0, 0, 0, 1, 0, 0, 0] |
| 4 | [0, 0, 0, 0, 1, 0, 0] |
| 5 | [0, 0, 0, 0, 0, 1, 0] |
| 6 | [0, 0, 0, 0, 0, 0, 1] |
| Liver or lung metastases |  |
| w/o | [1, 0, 0, 0] |
| To livers | [0, 1, 0, 0] |
| To lungs | [0, 0, 1, 0] |
| Both | [0, 0, 0, 1] |
| Peritoneum metastasis |  |
| w/o | [1, 0] |
| w/ | [0, 1] |
| Metastatic lymph nodes |  |
| w/o | [1, 0, 0] |
| No fusion | [0, 1, 0] |
| Fusion | [0, 0, 1] |
| Location of metastatic lymph nodes* |  |
| w/o | [1, 0, 0, 0, 0] |
| NO. 1-12 | [0, 1, 0, 0, 0] |
| NO. 13-20 | [0, 0, 1, 0, 0] |
| Med. or Virchow | [0, 0, 0, 1, 0] |
| Others | [0, 0, 0, 0, 1] |

Location of metastatic lymph nodes:

w/o: no metastatic lymph nodes

NO. 1-12: existing focal lymph nodes metastases

NO. 13-20: existing M-stage lymph nodes metastases in the peritoneum or retroperitoneum

Med. or Virchow: the presence of metastases in mediastinal or supraclavicular lymph nodes

Others: rare distant lymph node metastases (e.g., axillary, paracervical, inguinal, etc.)

* The categorization of metastatic lymph nodes is multi-label. When multiple labels apply, the final encoding will be a composite of the corresponding one-hot encodings. For instance, if a sample is identified as both NO. 1-12 and Med. or Virchow, the resulting embedding would be [0, 1, 0, 0, 0] | [0, 0, 0, 1, 0] = [0, 1, 0, 1, 0].

Table S13**. The patient information parameterless encoder.**

This pre-defined parameterless encoder was used to map patient information into embeddings.

| Characteristic | Embedding |
| --- | --- |
| Age (years) |  |
| $\boldsymbol{\leq}$60 | [1, 0] |
| $\boldsymbol{>}$60 | [0, 1] |
| Sex |  |
| Female | [1, 0] |
| Male | [0, 1] |
| Tumor site |  |
| GEJ | [1, 0] |
| Non-GEJ | [0, 1] |
| Anatomic position* |  |
| Stomach | [1, 0, 0, 0, 0, 0, 0, 0, 0, 0, 0, 0, 0] |
| Liver | [0, 1, 0, 0, 0, 0, 0, 0, 0, 0, 0, 0, 0] |
| Lung | [0, 0, 1, 0, 0, 0, 0, 0, 0, 0, 0, 0, 0] |
| Ovary | [0, 0, 0, 1, 0, 0, 0, 0, 0, 0, 0, 0, 0] |
| Peritoneum | [0, 0, 0, 0, 1, 0, 0, 0, 0, 0, 0, 0, 0] |
| Brain | [0, 0, 0, 0, 0, 1, 0, 0, 0, 0, 0, 0, 0] |
| Spleen | [0, 0, 0, 0, 0, 0, 1, 0, 0, 0, 0, 0, 0] |
| Adrenal gland | [0, 0, 0, 0, 0, 0, 0, 1, 0, 0, 0, 0, 0] |
| Pancreas | [0, 0, 0, 0, 0, 0, 0, 0, 1, 0, 0, 0, 0] |
| Bone | [0, 0, 0, 0, 0, 0, 0, 0, 0, 1, 0, 0, 0] |
| Regional LNM | [0, 0, 0, 0, 0, 0, 0, 0, 0, 0, 1, 0, 0] |
| Distant LNM | [0, 0, 0, 0, 0, 0, 0, 0, 0, 0, 0, 1, 0] |
| Abdominal wall | [0, 0, 0, 0, 0, 0, 0, 0, 0, 0, 0, 0, 1] |
| Degree of differentiation |  |
| Poorly | [1, 0, 0] |
| Moderately | [0, 1, 0] |
| Well | [0, 0, 1] |
| Lauren type |  |
| Intestinal | [1, 0, 0] |
| Diffused | [0, 1, 0] |
| Mixed | [0, 0, 1] |
| Treatment lines |  |
| 1 | [1, 0] |
| 2 or more | [0, 1] |
| Time to start treatment |  |
| 2007-2012 | [1, 0, 0] |
| 2012-2017 | [0, 1, 0] |
| 2017-2022 | [0, 0, 1] |

* The categorization of anatomic position is multi-label. When multiple labels apply, the final encoding will be a composite of the corresponding one-hot encodings. For instance, if a sample has several lesions in the stomach, liver, peritoneum, and adrenal gland, the resulting embedding would be [1, 0, 0, 0, 0, 0, 0, 0, 0, 0, 0, 0, 0] | [0, 1, 0, 0, 0, 0, 0, 0, 0, 0, 0, 0, 0] | [0, 0, 0, 0, 1, 0, 0, 0, 0, 0, 0, 0, 0]

| [0, 0, 0, 0, 0, 0, 0, 1, 0, 0, 0, 0, 0] = [1, 1, 0, 0, 1, 0, 0, 1, 0, 0, 0, 0, 0].

Table S14**.** **The patient information parameterless encoder in an external TCGA-STAD experiment.**

This pre-defined parameterless encoder was used to map patient information into embeddings.

| Characteristic | Embedding |
| --- | --- |
| Age (years) |  |
| $\boldsymbol{\leq}$60 | [1, 0] |
| $\boldsymbol{>}$60 | [0, 1] |
| Sex |  |
| Female | [1, 0] |
| Male | [0, 1] |
| Degree of differentiation |  |
| Poorly | [1, 0, 0] |
| Moderately | [0, 1, 0] |
| Well | [0, 0, 1] |
| Pathologic M |  |
| M0 | [1, 0] |
| M1 | [0, 1] |
| Pathologic N |  |
| N0 | [1, 0, 0, 0] |
| N1 | [0, 1, 0, 0] |
| N2 | [0, 0, 1, 0] |
| N3 | [0, 0, 0, 1] |
| Pathologic T |  |
| T0 | [1, 0, 0, 0, 0] |
| T1 | [0, 1, 0, 0, 0] |
| T2 | [0, 0, 1, 0, 0] |
| T3 | [0, 0, 0, 1, 0] |
| T4 | [0, 0, 0, 0, 1] |
| The lymph node examined count |  |
| 0–9 | [1, 0, 0, 0, 0, 0, 0, 0, 0, 0] |
| 10–19 | [0, 1, 0, 0, 0, 0, 0, 0, 0, 0] |
| 20–29 | [0, 0, 1, 0, 0, 0, 0, 0, 0, 0] |
| 30–39 | [0, 0, 0, 1, 0, 0, 0, 0, 0, 0] |
| 40–49 | [0, 0, 0, 0, 1, 0, 0, 0, 0, 0] |
| 50–59 | [0, 0, 0, 0, 0, 1, 0, 0, 0, 0] |
| 60–69 | [0, 0, 0, 0, 0, 0, 1, 0, 0, 0] |
| 70–79 | [0, 0, 0, 0, 0, 0, 0, 1, 0, 0] |
| 80–89 | [0, 0, 0, 0, 0, 0, 0, 0, 1, 0] |
| >90 | [0, 0, 0, 0, 0, 0, 0, 0, 0, 1] |
| The number of lymph nodes positive by he |  |
| 0–9 | [1, 0, 0, 0, 0, 0] |
| 10–19 | [0, 1, 0, 0, 0, 0] |
| 20–29 | [0, 0, 1, 0, 0, 0] |
| 30–39 | [0, 0, 0, 1, 0, 0] |
| 40–49 | [0, 0, 0, 0, 1, 0] |
| 50–59 | [0, 0, 0, 0, 0, 1] |
| Race |  |
| White | [1, 0, 0] |
| Asian | [0, 1, 0] |
| Black or African American | [0, 0, 1] |

Table S15**. The patient information parameterless encoder in an external TCGA-BRCA experiment.**

This pre-defined parameterless encoder was used to map patient information into embeddings.

| Characteristic | Embedding |
| --- | --- |
| Age (years) |  |
| $\boldsymbol{\leq}$60 | [1, 0] |
| $\boldsymbol{>}$60 | [0, 1] |
| Sex |  |
| Female | [1, 0] |
| Male | [0, 1] |
| Pathologic M |  |
| M0 | [1, 0] |
| M1 | [0, 1] |
| Pathologic N |  |
| N0 | [1, 0, 0, 0] |
| N1 | [0, 1, 0, 0] |
| N2 | [0, 0, 1, 0] |
| N3 | [0, 0, 0, 1] |
| Pathologic T |  |
| T0 | [1, 0, 0, 0, 0] |
| T1 | [0, 1, 0, 0, 0] |
| T2 | [0, 0, 1, 0, 0] |
| T3 | [0, 0, 0, 1, 0] |
| T4 | [0, 0, 0, 0, 1] |
| The lymph node examined count |  |
| 0–9 | [1, 0, 0, 0, 0, 0, 0, 0, 0, 0] |
| 10–19 | [0, 1, 0, 0, 0, 0, 0, 0, 0, 0] |
| 20–29 | [0, 0, 1, 0, 0, 0, 0, 0, 0, 0] |
| 30–39 | [0, 0, 0, 1, 0, 0, 0, 0, 0, 0] |
| 40–49 | [0, 0, 0, 0, 1, 0, 0, 0, 0, 0] |
| 50–59 | [0, 0, 0, 0, 0, 1, 0, 0, 0, 0] |
| 60–69 | [0, 0, 0, 0, 0, 0, 1, 0, 0, 0] |
| 70–79 | [0, 0, 0, 0, 0, 0, 0, 1, 0, 0] |
| 80–89 | [0, 0, 0, 0, 0, 0, 0, 0, 1, 0] |
| >90 | [0, 0, 0, 0, 0, 0, 0, 0, 0, 1] |
| The number of lymph nodes positive by he |  |
| 0–9 | [1, 0, 0, 0, 0, 0] |
| 10–19 | [0, 1, 0, 0, 0, 0] |
| 20–29 | [0, 0, 1, 0, 0, 0] |
| 30–39 | [0, 0, 0, 1, 0, 0] |
| 40–49 | [0, 0, 0, 0, 1, 0] |
| 50–59 | [0, 0, 0, 0, 0, 1] |
| Race |  |
| White | [1, 0, 0] |
| Asian | [0, 1, 0] |
| Black or African American | [0, 0, 1] |

**Supplementary references:**

1 Chen, Z. *et al.* propnet: Propagating 2D Annotation to 3D Segmentation for Gastric Tumors on CT Scans. Preprint at https://doi.org/10.48550/arXiv.2305.17871 (2023).

2 Wolff, A. C. *et al.* Human Epidermal Growth Factor Receptor 2 Testing in Breast Cancer: American Society of Clinical Oncology/College of American Pathologists Clinical Practice Guideline Focused Update. *J Clin Oncol*. **36**, 2105-2122, (2018).

3 Paszke, A. *et al.* Pytorch: An imperative style, high-performance deep learning library. Preprint at https://doi.org/10.48550/arXiv.1912.01703 (2019).

4 Robbins, H. & Monro, S. A stochastic approximation method. *The annals of mathematical statistics*, 400-407, (1951).

5 Tan, M. *et al.* in *Proceedings of the IEEE/CVF conference on computer vision and pattern recognition.* 2820-2828.

6 van Griethuysen, J. J. M. *et al.* Computational Radiomics System to Decode the Radiographic Phenotype. *Cancer Res*. **77**, e104-e107, (2017).

7 Deng, J. *et al.* in *2009 IEEE conference on computer vision and pattern recognition.* 248-255 (Ieee).

8 Vaswani, A. *et al.* Attention is all you need. *Advances in neural information processing systems*. **30**, (2017).

9 Ba, J. L., Kiros, J. R. & Hinton, G. E. Layer normalization. Preprint at https://doi.org/10.48550/arXiv.1607.06450 (2016).

10 Isensee, F. *et al.* nnU-Net: a self-configuring method for deep learning-based biomedical image segmentation. *Nat Methods*. **18**, 203-211, (2021).

11 Katzman, J. L. *et al.* DeepSurv: personalized treatment recommender system using a Cox proportional hazards deep neural network. *BMC Med Res Methodol*. **18**, 24, (2018).

12 Chen, T., Kornblith, S., Norouzi, M. & Hinton, G. in *International conference on machine learning.* 1597-1607 (PMLR).
