# Supplementary material for: Predicting gastric cancer response to anti-HER2 therapy or anti-HER2 combined immunotherapy based on multi-modal data
Source: Signal Transduct Target Ther. 2024 Aug 26;9:222. doi: 10.1038/s41392-024-01932-y (PMC11345439; doi:10.1038/s41392-024-01932-y)
Supplement: Supplementary file 2 — Source code [file 41392_2024_1932_MOESM2_ESM.pdf]

The source code is available at the Google drive:
https://drive.google.com/file/d/1JVDGEI8Kb5LKivJkvwYCJJ8775iiDH-U/view?usp=sharing

or Baidu drive (code: c5df):
https://pan.baidu.com/s/19F-U5rOpRvaATdALHkiHiA?pwd=c5df
